# Environmental Xenoestrogens Super-Activate a Variant Murine ER Beta in Cholangiocytes

**DOI:** 10.1093/toxsci/kfw234

**Published:** 2016-12-24

**Authors:** Stephanie K. Meyer, Philip M. E. Probert, Anne K. Lakey, Alastair C. Leitch, Lynsay I. Blake, Paul A. Jowsey, Martin P. Cooke, Peter G. Blain, Matthew C. Wright

**Affiliations:** * Institute Cellular Medicine, Level 4 Leech, Newcastle University, Newcastle Upon Tyne NE24HH, UK;; † Health Protection Research Unit, Wolfson Building, Newcastle University, Newcastle Upon Tyne NE2 4AA, UK;; ‡ Institute for Sustainability, The Key Building, Newcastle University, Newcastle upon Tyne NE4 5TQ, UK;; § School of Civil Engineering and Geosciences, Drummond Building, Newcastle University, Newcastle upon Tyne NE1 7RU, UK

**Keywords:** estrogen, liver, cholestasis, biliary, ER, nuclear receptor.

## Abstract

High systemic levels of oestrogens are cholestatic and primary biliary cholangitis (PBC)—which is characterized by hepatic ductular inflammation—is thought to be triggered by exposure to xenobiotics such as those around landfill sites. Xenoestrogens may be a component of this chemical trigger. We therefore hypothesized that xenoestrogens are present at higher levels in the proximity of landfill sites. To test this hypothesis, soil samples were collected, extracts prepared and biological oestrogenic activity examined using cell-based reporter gene assays. Extracts from several sample sites around a landfill site contained a chemical(s) which activated the human ERα in a dose-dependent manner. Extracts from 3 separate control sampling sites were absent of any detectable activity. The mouse ERα and 2 variant mouse ERβ cDNAs were cloned and extracts from sample sites around a landfill site also activated these receptors. One variant murine ERβ was constitutively active when expressed in cholangiocytes, was readily inactivated by ICI182780 and activated in a dose-responsive, ICI182780-inhibitable manner by oestrogen. However, when this receptor was activated by extracts from landfill site soils, ICI182780 failed to antagonize activation. ERβ was readily detectable in murine cholangiocytes and exposing mice acutely to a pooled ER activating soil extracts also gave rise to a mild cholestatic injury. These data indicate that the environment around landfill sites may contain higher levels of xenoestrogens; that these chemicals have “super-activating” characteristics with a variant ERβ and therefore these chemicals could be a component of a xenobiotic insult that triggers PBC.

A variety of man-made chemicals released into the environment by anthropogenic activity (eg, pesticides) have been shown to act as endocrine disruptors and have been proposed to be responsible for a spectrum of adverse effects in wildlife and man, from malformations in the male genital tract; decreased sperm quality; neuroendocrinological, behavioural and metabolic effects to cancer (Alonso-Magdalena *et al.*, 2011; Mattison *et al.*, 2014; Soto and Sonnenschein, 2010). Oestrogens (estrogens) are a class of steroid hormones initially defined on the basis of their ability to bind and activate the oestrogen receptors (ERs) (Bondesson *et al.*, 2015). The main oestrogen in humans is 17β oestradiol (E2) although other circulating oestrogens having biological activity on the human ERs include oestrone (E1) and oestriol (E3) (Bondesson *et al.*, 2015).

Oestrogens are primarily produced in the ovaries or testes from testosterone and androstenedione (to produce E2 and E1, respectively) through metabolism by aromatase, and their major biological function in women is to regulate the variety of physiological changes associated with female reproduction (in sexually mature women) (Bondesson *et al.*, 2015). These changes most obviously include developmental changes in reproductive-relevant tissues such as the uterus and breast. However, oestrogens also regulate metabolic changes associated with actions on other tissues eg, hepatic synthesis of sex hormone binding globulin (Hammond, 2011); hepatic metabolic effects (Mauvais-Jarvis *et al.*, 2013). The majority of effects of oestrogens are widely considered to be mediated through their interaction with ERs. ERs are members of the nuclear receptor gene superfamily of Zn finger-containing transcription factors which—in response to interactions with specific ligands—modulate the transcription of genes regulated by response element sequences that bind the nuclear receptor complexes (Bondesson *et al.*, 2015). Thus, the sequence of the response element is a major determinant in conferring regulation by a particular ligand via its interacting nuclear receptor.

Two ER genes are present within the human genome - ERα (NR3A1) and ERβ (NR3A2) and their products are both activated by E2 (Bondesson *et al.*, 2015). ERα is expressed at high levels in uterine, ovarian, pituitary gland, vas deferens and adipose tissues (Nuclear Receptor Signaling Atlas www.nursa.org/; last accessed November 18, 2016) and the primary role of ERα is the regulation of sexual reproduction, as exemplified in ERα knock out mice—females are infertile and males have decreased fertility (Lubahn *et al.*, 1993). ERβ expression is highest in the ovary, lung, epididymis, prostate, colon and specific regions of the brain (Nuclear Receptor Signalling Atlas www.nursa.org/). ERβ knockout mice develop relatively normally and have normal fertility (Krege *et al.*, 1998). Additional receptors for oestrogens have been identified which expand the potential mechanism by which oestrogens and xenoestrogens may affect cells. These include other nuclear receptors such as the oestrogen-related receptors which bind some isoflavones, but not E2 (Suetsugi *et al.*, 2003) and the constitutive androstane receptor in both mouse (Kawamoto *et al.*, 2000) and man (Koh *et al.*, 2012). In addition, the non-genomic effects of oestrogens may be mediated via ERs (or potentially other oestrogen-binding nuclear receptors) located on membranes and acting in a relative rapid (ie, minutes rather than the hours required for transcriptional changes), non-canonical manner (Rainville *et al.*, 2015). Oestrogen binding proteins that are structurally unrelated to nuclear receptors (eg, GPER1, also known as GPR30), are often membrane associated and have also been identified to mediate non-genomic rapid effects of oestrogens (such as kinase activation and changes in intracellular calcium) (Rainville *et al.*, 2015).

Xenoestrogens are exogenous chemicals which mimic the activity of oestrogens through their interaction with cellular components that interact with endogenous oestrogens and through these interactions modulate the normal levels and/or endocrine activity of oestrogens. Xenoestrogens include chemicals naturally present in our diet eg, isoflavones (Kuiper *et al.*, 1998) and a variety of synthetic man-made chemicals eg, bisphenol A (Laws *et al.*, 2000). Thus, a xenoestrogen may be a chemical that interacts with receptor proteins mediating the biological effect of oestrogens as hormones. However, other mechanisms of xenoestrogen action also exist, related to any biologically relevant alteration in oestrogen bioavailability. Thus, a chemical that causes alterations in oestrogen synthesis, sequestration, dynamic activity, metabolism and/or excretion could be defined as a xenoestrogen.

The liver is considered a hormonal target for oestrogens via ERα (Ahlbory-Dieker *et al.*, 2009) and plays a major role in determining the circulating levels of oestrogens via metabolic conversion of oestrogens to inactive products (Bondesson *et al.*, 2015; Tsuchiya *et al.*, 2005; Ziegler et al., 2015). Its importance is exemplified by the feminisation that occurs in men with chronic liver disease due, in part, to impaired hepatic oestrogen metabolism and clearance (Burra, 2013). The liver is also a target organ for the toxic effects of oestrogens—the classic response being that of a disruption of bile flow and/or alteration in bile constituents (cholestasis). Cholestasis leads to an accumulation of bile acids in the liver, which is toxic and results in liver cell necrosis and apoptosis as well as systemic adverse effects eg, pruritis (Woolbright and Jaeschke, 2012). In susceptible individuals, the elevations in circulating oestrogens in pregnancy or through use of contraceptives can be sufficient to lead to hepatic failure and death in the absence of liver transplantation (Ozkan *et al.*, 2015). In terms of natural oestrogens and adverse hepatic effects, Stieger *et al.* (2000) proposed that oestrogen and/or its hepatic metabolites inhibit the activity of bile acid and drug transporters to initiate cholestasis. However, in 2006, Negishi and co-workers elegantly demonstrated that the expression of many bile acid and drug transporters are transcriptionally repressed by ERα activation, an effect lost in ERα null mice (Yamamoto *et al.*, 2006). These latter data therefore suggest that xenoestrogens having a potential adverse effect in the liver may be identified by their interaction with the ERα.

Primary Biliary Cholangitis (PBC) is a chronic liver disease thought to be of an autoimmune aetiology due to the presence of antibodies to mitochondrial proteins in the majority of PBC patients (Dyson *et al.*, 2015; Griffiths and Jones, 2014). At present, treatment options for PBC are limited and there are no proven strategies to prevent the onset of the disease in individuals known to be at risk of the disease (including the daughters of mothers with the disease who have a 35-fold increased risk of developing the disease). PBC is triggered in genetically predisposed individuals through exposure to an unknown chemical(s). Much of the epidemiology over the last few decades has linked PBC to chemicals such as the use of hair dyes; to proximity to toxic landfill sites and to areas of heavy mining (Ala *et al.*, 2006; Prince *et al.*, 2001; Smyk *et al.*, 2010). Although some chemicals have been shown to trigger a PBC-like effect in mouse models (Wakabayashi *et al.*, 2008; Walden *et al.*, 2008), actual chemical(s) trigger in the environment and causative mechanism(s) for the disease are unknown.

Previous investigations have suggested that only ERα is expressed in hepatocytes whereas both ERα and ERβ are expressed in cholangiocytes and markedly upregulated in disease settings. It has been considered that through actions on both (ERα) and (ERβ) subtypes, oestrogens regulate the expression of growth factors and cytokines, thereby modulating the proliferative response of cholangiocytes to damage (Alvaro *et al.*, 2004, 2006).

Given the widespread concern regarding man-made xenoestrogen chemicals and adverse health effects, we hypothesized that the environment around landfill sites contains a variety of xenoestrogens and that chronic exposure may give rise to some of the effects associated with endocrine disruptors, such as cholestasis. To test this hypothesis, soil samples were taken from around a currently active peri-urban landfill site positioned upon an area of historic mining (and from 3 separate control sampling sites), chemicals were extracted into an ethanol solvent and the presence of oestrogenic chemicals tested in biological assays (human and mouse ER-luciferase reporter gene assays).

In this article, we demonstrate that several landfill sample sites contained oestrogenic chemicals, and in contrast to E2, behaved as irreversible agonists of a murine ERβ variant in a pancreatohepatobiliary cell context. Acute exposure of mice to a pooled oestrogenic environmental sample also resulted in evidence of cholestasis.

## MATERIALS AND METHODS

The mouse cholangiocyte cell line 603B was a gift from Dr Yedidya Saiman, Mount Sinai School of Medicine, New York. The mouse pancreatic ductal cell line LTPA was originally obtained from the American Type Culture Collection (ATCC, catalog CRL-2389, Manassas, Virginia) and is an epithelial cell line derived from a spontaneous pancreatic adenocarcinoma taken from a 12-month old female Lt/Sv mouse. When injected subcutaneously into Swiss nu/nu mice, LTPA cells form ductular structures (ATCC, catalog CRL-2389, Manassas, Virginia). Although these cells are not of hepatic origin, the pancreatic and biliary ducts are physiologically connected and have a shared developmental origin (Probert *et al.*, 2015). These cells were used as they supported ERα reporter gene assays in terms of responsiveness when compared with baseline expression and in terms of good repeatability. The human breast cancer cell line MCF-7 was a kind gift from Dr Katherine Rennie, Newcastle University. The 293 (HEK293) cell line was purchased from the European Collection of Cell Cultures (ECACC, Porton Down, UK). E2, EE and ICI182780 were purchased from Sigma (Poole, UK).

### 

#### 

##### Soil sample collection and extract preparation

Soil samples (of approximately 0–5 cm in depth) were collected around the perimeter (within 200 m) to an active landfill site (located within a peri-urban area) which does not have a license to accept hazardous materials. In addition, 3 control soil samples were similarly collected from 3 separate sites. One sample was obtained from the University farm in rural Northumberland at a site with controlled fertilizer regime for the last 130 years and the remaining 2 control samples were obtained from gardens in urban areas in the region. Extraneous vegetable matter and stones removed, manually homogenized and stored at 5°C until downstream use. 125 g soil was sonicated in 300mls methanol for 10 min, followed by addition of a further 100 ml of solvent and sonication for a further 10 min prior to filtration with 25 µm filters and collection of filtrate. Filtrates were evaporated in a rotary evaporator and then blown down to near dryness under a stream of nitrogen prior to addition of 30 ml of ethanol. The solvated extracted chemicals were separated from any precipitate and stored at −20°C.

##### Recombinant DNA cloning

The mouse (henceforth prefixed with an m; human receptor prefixed with an h) mERα and mERβ variant 1 (m mERβv1) and variant 2 (mERβv2) cDNAs were amplified from mRNA isolated from mouse uterus and mouse ovary, respectively, using the following primers, mERacloning, US, 5′- CGCCGAATTCCACTTACCATGACCATG-3′ DS, 5′- CCTGGAAGCTTTCAGATCGTGTTGGGG-3′; mERßcloning, US, 5′- CCGTGAATTCCTGAGAGCATCATGTCCA-3′ DS, 5′- TCCGCCTTAAGCCTGGCCGTCACTG-3′ to give *Hind*III and *EcoR*I restriction sites for mERα and *Afl*II and *EcoR*I sites for mERβ cDNA products. The PCR products were initially cloned into a pCR-blunt vector using the Zero Blunt PCR Cloning Kit (Life technologies) prior to sub-cloning into pcDNA3.1 restricted with the appropriate restriction enzymes. Constructs were screened for correct insertion by restriction digest analyses and sequence integrity by DNA sequencing.

##### Cell line culture

603B, MCF-7 and 293 cells were cultured in low glucose Dulbecco’s Modified Eagles Medium (Sigma, Dorset, UK), supplemented with 10% (v/v) foetal bovine serum (Sigma) and 80 U/ml of penicillin and streptomycin. LTPA cells were cultured in the above medium further supplemented with 0.1 mM non-essential amino acids (Gibco, Life technologies, Paisley, UK) and 1 mM sodium pyruvate (Gibco, Life technologies). All cell lines were maintained in a humidified atmosphere at 37 °C in 5% CO_2_ in air.

##### RT-PCR

Total RNA was purified using Trizol (Invitrogen, Paisley, UK). RT-PCR was carried out as previously outlined (Probert *et al.*, 2014) and using primer sequences given in Table 1.

**TABLE 1:** Primers used for Cloning and RT-PCR

Oligo ID	5′-3′ sequence	Annealing (°C)	Comments
mERαUS	AAGGGCAGTCACAATGAACC	59	Will amplify mouse ERα (NM_007956) cDNA sequence of 155 bp
mERαDS	GCCAGGTCATTCTCCACATT		
mERβUS	GGGTGAAGGAGCTACTGCTG	59	Will amplify mouse ERβ transcript variants 1 and 2 (NM_207707 and NM_010157) cDNA sequence of 576 and 522 bp, respectively
mERβDS	GTGTCAGCTTCCGGCTACTC		
mGAPDHUS	TGACATCAAGAAGGTGGTGAAG	55	Will amplify mouse (NM_008084) glyceraldehyde 3 phosphate dehydrogenase cDNA sequence of 243 bp
mGAPDHDS	TCTTACTCCTTGGAGGCCATGT		
mCK19US	GAGATCATGGCCGAGAAGAA	55	Will amplify mouse cytokeratin 19 (NM_008471.2) cDNA sequence of 72 bp
mCK19DS	GGTGTTCAGCTCCTCAATCC		
mVimentinUS	GTGGCTCCGGCACATCGAGC	56	Will amplify mouse vimentin (NM_011701.4) cDNA sequence of 226 bp
mVimentinDS	GCGTCGGCCAGCGAGAAGTC		
mCYP2E1US	GTGTTCCGAGGATATGTCATC	56	Will amplify mouse CYP2E1 (NM_021282.2) cDNA sequence of 223 bp
mCYP2E1DS	AAAGCAGAAACAGTTCCATGC		
mERaCloningDSHindIII	CCTGGAAGCTTTCAGATCGTGTTGGGG	72	Will amplify mouse ERα transcript variants 1, 2 and 3 (NM_007956.5, NM_001302531.1, NM_001302532.1) cDNA sequence of 1829 bp (2-step PCR)
mERaCloningUSEcoRI	CGCCGAATTCCACTTACCATGACCATG		
mERβUScloning	TCCGCCTTAAGCCTGGCCGTCACTG	65	Will amplify mouse ERβ transcript variants 1 and 2 (NM_207707 and NM_010157) cDNA sequence of 1744 and 1690 bp, respectively
mERβDScloning	TCCGCCTTAAGCCTGGCCGTCACTG		

##### Transfection and reporter gene assays

603B and LTPA cells were transiently transfected in 24-well plates with 0.25 μg total DNA per well (pcDNA3.1 expression vector encoding the cDNAs for either the mERα, mERβv1, mERβv2, mouse c/EBPβ or an empty construct (ie, pcDNA3.1); an oestrogen-responsive reporter gene luciferase construct, either (ERE)_3_-pGL3promoter (Axon *et al.*, 2012) or 3XERE TATA Luc (Hall and McDonnell, 1999; obtained via Adgene plasmid no. 11354) and a control plasmid (RL-TK) encoding the Renilla luciferase protein under the regulation of a constitutive thymidine kinase promoter to control for transfection efficiency between wells at a ratio of 6, 6, 1) using Effectene reagent (Qiagen, Manchester, UK), according to the manufacturer’s instructions. The mERα failed to transactivate the (ERE)_3_-pGL3promoter in response to E2 treatment (data not included) and therefore the 3XERE TATA Luc previously shown to respond to mouse ERα activation (La Sala *et al.*, 2010) was employed. Twenty-four hours after transfection, cells transfected with the pcDNA3.1 constructs coding for the mERα or mERβv1 were, where applicable, pre-treated with the ER antagonist ICI182780 for 6 h before being treated with oestrogens or potential xenoestrogens from 1000-fold concentrated stocks in DMSO or PBS. Since the mERβv2 exhibited near complete constitutive activity *in vitro*, cells transfected with the pcDNA3.1 construct encoding the mERβv2 were pre-treated with the ER antagonist ICI182780 for 6 h followed by 5 wash steps with large volumes of sterile PBS to reduce the levels of antagonist. Cells were then treated with oestrogens or potential xenoestrogens from 1000-fold concentrated stocks in DMSO or PBS in the absence, and where indicated, in the presence of ICI182780. Following treatment for 24 h, Firefly and Renilla luciferase activities were determined using a Dual-Glo luciferase assay kit (Promega) as previously described in Axon *et al.* (2012). Charcoal-stripped serum was not used (to reduce serum oestrogens) unless explicitly indicated since in our hands, avoiding this procedure produced high readout-to-background data with reduced intra-experimental replicate variability (although inter-experimental maximum fold induction was more variable). When charcoal-stripped serum was used, sera were prepared as previously outlined (Axon *et al.*, 2012), in some cases in conjunction with phenol red-free culture medium.

##### Cholangiocyte isolation and culture

Male C57Bl/6 wild type mice were terminated by cervical dislocation. The liver was removed and 6–7 pooled, minced using a scalpel in 25 ml Hank’s balanced salt solution plus calcium (HBSS+, 0.14M NaCl, 5.4 mM KCl, 0.34 mM Na_2_HPO_4_ 12H_2_O, 0.44 mM KH_2_PO_4_, 5.6 mM glucose, 6 mM HEPES, 0.35 g/l NaHCO_3_ and 1 mM CaCl_2_) supplemented with 1 mg/ml collagenase type 1A (Sigma) and 80 μg/ml DNAse I (Sigma). After 30–45 min incubation at 37 °C, the digest was filtered through a 125 μm Nybolt mesh and the resultant cell suspension made up to 100 ml with HBSS+. Cells were pelleted by centrifugation at 600 g for 5 min at room temperature. This washing step was repeated twice and the final cell pellet re-suspended in 12 ml HBSS+. Mouse cholangiocytes were semi-purified by density gradient centrifugation as previously described using percoll density centrifugation (Joplin *et al.*, 1989) (without additional anti-HEA125 affinity purification since the antibody did not immunoreact with its murine ortholog in our hands and offered no improvements in purity or yield). Percoll (GE Healthcare) was diluted 9:1 (v/v) with 10× PBS (1.37M NaCl, 27 mM KCl, 100 mM phosphate pH 7.4). This stock was diluted 1:2 (v/v) with 1× PBS (137 mM NaCl, 2.7 mM KCl, 10 mM phosphate pH 7.4) and 3 ml was layered onto 3 mls of stock diluted 1:0.3 (v/v) with 1× PBS in a 13 ml sterile falcon tube. In total, 3 ml of cells suspension (in HBSS+) was then layered onto the top of each percoll layer followed by centrifugation at 670 g for 30 min at 80% acceleration and 0% deceleration in a swing out rotor. The lower percoll/percoll interface was enriched in cholangiocytes and these were collected, added to 3 volumes of 1×PBS, pelleted by centrifugation at 600 g for 5 minutes and re-suspended in culture media (1:1 [v/v] DMEM, Hams F12 medium supplemented with 10% (v/v) FBS, 2 mM glutamine, 100 U/ml penicillin, 100 μg/ml streptomycin, 10 ng/ml epidermal growth factor, 0.248 IU/ml Insulin, 2 µg/ml hydrocortisone, 10 ng/ml cholera toxin, 2nM tri-iodo-L-thyronine and 5 ng/ml hepatocyte growth factor. Cells were seeded onto plastic culture dishes and maintained in a humidified atmosphere at 37 °C in 5% CO_2_ in air.

##### Immunocytochemistry

Cells were fixed with 4% formaldehyde (Sigma) in PBS for 10 min followed by permeabilization with 0.2% (w/v) Triton in 1× PBS for 15 min. Non-specific protein binding was blocked by incubation with 5% goat serum in 1× PBS-T (PBS + 0.02% w/v Tween 20) for 20 min at room temperature. Cells were incubated with anti-cytokeratin 19 (CK19) (Abcam, ab 84632) plus anti-ERα (Abcam, ab2746) or anti-ERβ (Abcam, ab288) primary antibodies in blocking buffer at 4 °C overnight. Following three 10-min washes in PBS-T, cells were incubated with the appropriate secondary antibodies (Cell signaling, 4412S and 4414S) in blocking buffer for 45 min in the dark. Cell nuclei were stained with DAPI prior to mounting using Fluoroshield mountant (Sigma).

##### Western blotting

Total protein was isolated from cell and tissue samples using RIPA buffer (150 mM sodium chloride, 1.0% Triton X-100, 0.5% sodium deoxycholate, 0.1% sodium dodecyl sulfate, 50 mM Tris, pH 8.0), 30 min agitation at 4 °C. 4× LDS sample buffer (Life Technologies) and 5% v/v 2-mercaptoethanol was added to cell lysates, samples were boiled for 5 min at 95 °C and the genomic DNA sheared by sonication. Proteins were separated using pre-cast 4–12% Bis-Tris polyacrylamide gels (NuPAGE, Life Technologies) and then transferred to nitrocellulose membranes using the iBlot device (Life technologies). Membranes were probed using standard protocols with the following primary antibodies, Rabbit polyclonal antibodies to ERα (Santa Cruz Biotechnology, sc-7207, 1, 500 dilution) and ERβ (Abcam, ab3576, 1:1000 dilution). Anti-GAPDH antibody was purchased from Cell Signaling (2118S, 1:5000 dilution). Appropriate HRP-conjugated anti-IgG antibodies were used for detection using the ECL reagent (GE Healthcare). Protein bands were visualized using a Syngene G, Box gel documentation system.

##### Animal study

Male adult C57Bl/6 wild type mice were purchased from Charles River (Kent, UK) and housed in accordance with UK Home Office regulations. Males were chosen over females because the female oestrus cycle results in more variable levels of endogenous oestrogens in test groups of animals, which would result in more mice per test group being required to assess any potential effects of a xenoestrogen. On a 3Rs basis, it was considered more ethical to test any potential effects of xenoestrogens on as few mice as possible, and so male mice were selected. Food and water for animals were available ad libitum and conditions were kept at 23 ± 1 °C on a 12-h light/12-h dark cycle at 47% humidity. Mice were exposed to combined and concentrated soil extracts which have been shown to exhibit oestrogenic activity in a reporter gene assay or to combined soil extracts from the 3 control sampling sites without any detectable oestrogenic activity by single intraperitoneal injection. Other groups received either ethinyloestradiol (EE, Sigma, Poole, UK) at 0.5 mg/kg bodyweight in 90% olive oil/10% ethanol vehicle or vehicle alone. For preparation of the extracts, combined oestrogen positive or oestrogen negative soil ethanol extracts were dried down under a stream of nitrogen and re-suspended in 90% olive oil/10% ethanol (v/v) at half the original volume. All animals were dosed at 20 ml per kg bodyweight. Mice were terminated 24 h postinjection and their blood and livers were collected. Experiments were performed under a UK Home Office licence with Local Ethics Committee approval.

##### Clinical chemistry And histology

Blood was collected at termination, serum prepared and clinical chemistry (ALP, ALT) determined as described (Marek *et al.*, 2005). Following dissection, livers were fixed in 10% PBS buffered formalin for 24 h before paraffin embedding and sectioning at 4 μm. Tissue sections were stained with Haematoxylin and Eosin as previously described in Probert *et al.* (2014).

## RESULTS

### 

#### Extracts from Soil Samples around a Landfill Site Contain Activators of the Human ERα

MCF-7 cells were transiently transfected with the (ERE)_3_-pGL3promotor using a previously validated protocol for screening hERα transcriptional activity (Axon *et al.*, 2012) and treated with ethanol extracts from soil samples taken from around an urban landfill site (landfill site soil samples). The ethanol solvent stock used in the soil extraction procedure was used as solvent control. Identical procedures were used to obtain extracts from 3 different sites not in close proximity to an urban landfill site (control site soil samples).

**FIG. 1:**
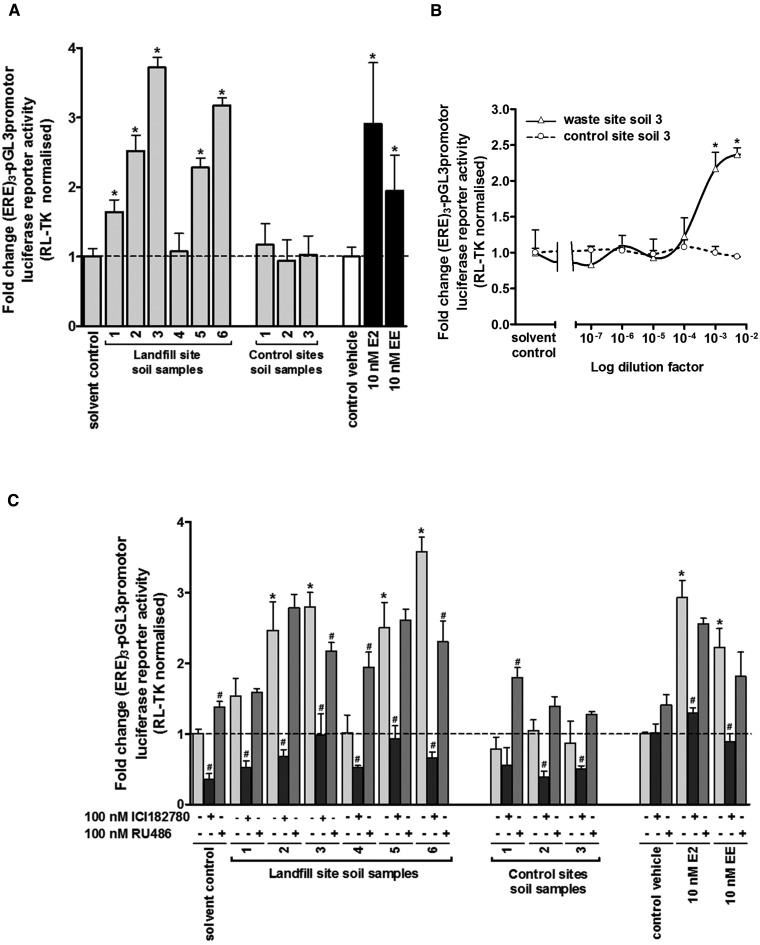
Extracts of soil samples in close proximity to a landfill site contain a chemical(s) that activate the human ERα. **A**, luciferase reporter gene (ERE)_3_-pGL3promoter assay in MCF7 cells. Cells were transfected as outlined in the methods section and treated with 0.5% v/v ethanol extracts, E2 or EE for 1 day prior to analysis. **B**, concentration-dependent luciferase reporter gene assay in MCF7 cells treated essentially as outlined in A with the indicated dilution of landfill site soil extract 3 or control extract 3. **C**, MCF7 were cells treated essentially as outlined in A except cells were pre-treated for 5 h with the indicated nuclear receptor antagonist or solvent vehicle as control prior to exposure to soil sample extracts, E2 or EE for 1 day. Data are the mean and SD normalized luciferase activity from 3 entirely separate determinations of the same conditions performed within the same experiment, typical of at least 3 separate experiments performed at different times, significantly different versus *ethanol solvent control or #antagonist-free treated cells using the Student’s *t* test (2-tailed), *P* < .05.

Figure 1A demonstrates that media supplemented with ethanol extracts effectively diluted by medium by a factor of 1000 (ie, constituting a final 0.1% v/v) resulted in significant increases in normalized luciferase reporter gene expression in several sampling sites around the landfill site and to levels similar or greater than that induced by treatment with a biological fully saturating 10nM E2 or 10 nM EE. In contrast, no significant increase in normalized luciferase reporter gene expression was observed when cells were treated with similarly diluted ethanol extracts from 3 control site soil samples.

Normalized luciferase reporter gene expression was increased by selected landfill site ethanol extracts in a dose–response manner (Figure 1B) and was inhibited by the ER antagonist ICI182780 but not by RU486 (an antagonist for other nuclear receptors) (Figure 1C). These data therefore indicate that ethanol extracts from soil samples in close proximity to an urban landfill site contained a chemical(s) that was capable of producing a readily detectable activation of the hERα in a cell-based biological reporter gene assay.

#### Extracts from Soil Samples around a Landfill Site Activate the Murine ERs

**FIG. 2:**
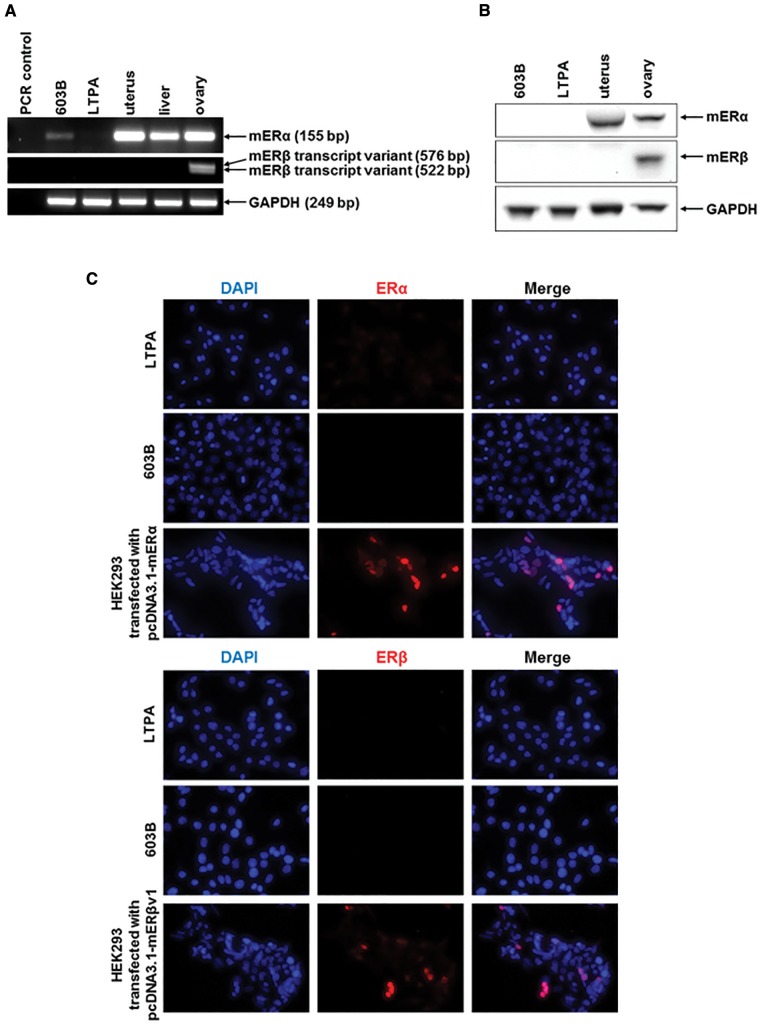
Expression of murine ERs in murine pancreatohepatobiliary cell lines. **A**, RT-PCR analysis for the indicated mRNA transcript in mouse 603B or LTPA cell lines and comparison to intact mouse tissues. **B**, Western blot for the indicated protein in ductal cell lines or the indicated murine tissue (20 μg total protein/lane). **C**, immunocytochemical analysis for ER expression in murine pancreatobiliary cell lines and in HEK293 cells transfected with an expression vector encoding the indicated ER cDNA.

The mouse was considered the preferred animal model in which to study the potential effects of xenoestrogens (given, for example, the availability of a variety of transgenic lines in follow on studies). Since cholangiocytes on a background of disease express both ERs in rats and man (Alvaro *et al.*, 2000, 2002a,b, 2004, 2006), cholangiocytes isolated from mouse liver were initially examined as the cell type for examining ER activation. However, mouse cholangiocytes resisted transfection (data not shown) and therefore cell lines were used as an alternative. Only 2 cell lines were available, LTPA and 603B cells. Figure 2A demonstrates however, that mER mRNA transcripts were not readily detectable in these cell lines, with likely a very low expression of mERα mRNA in 603B cells only. In any case, ER proteins were undetectable in both cell lines (Figs. 2B and C). In order to study ER function in these cells, the protein encoding murine mER cDNAs were therefore cloned and ligated into expression vectors to facilitate their ectopic expression.

**FIG. 3:**
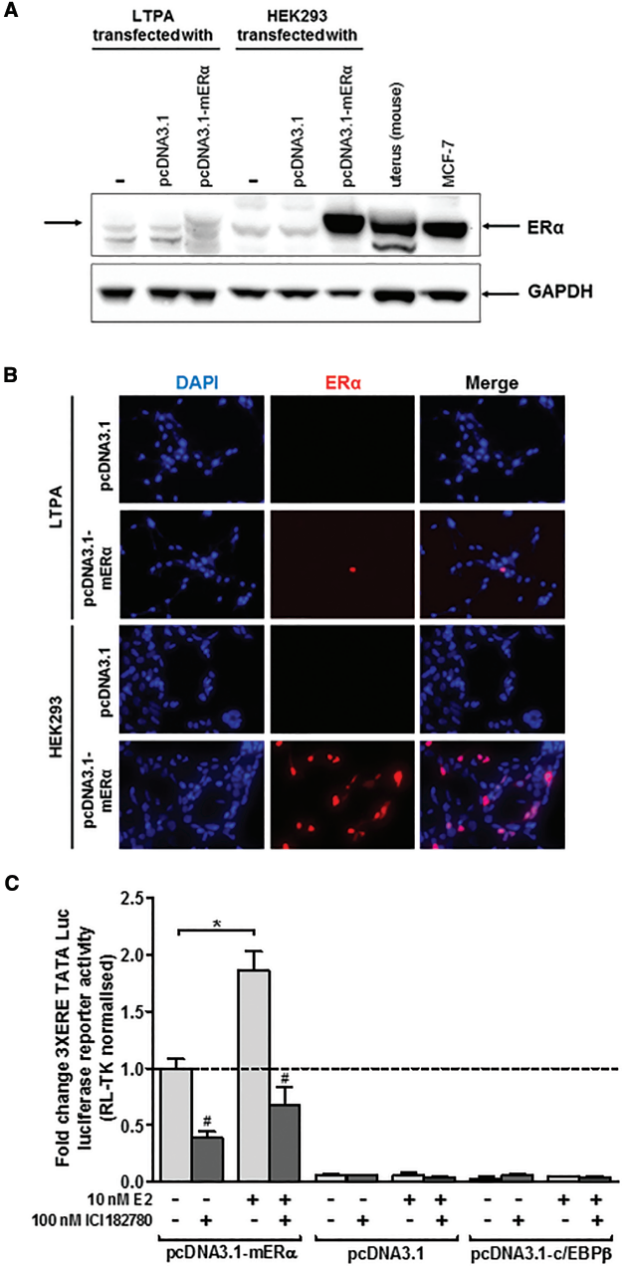

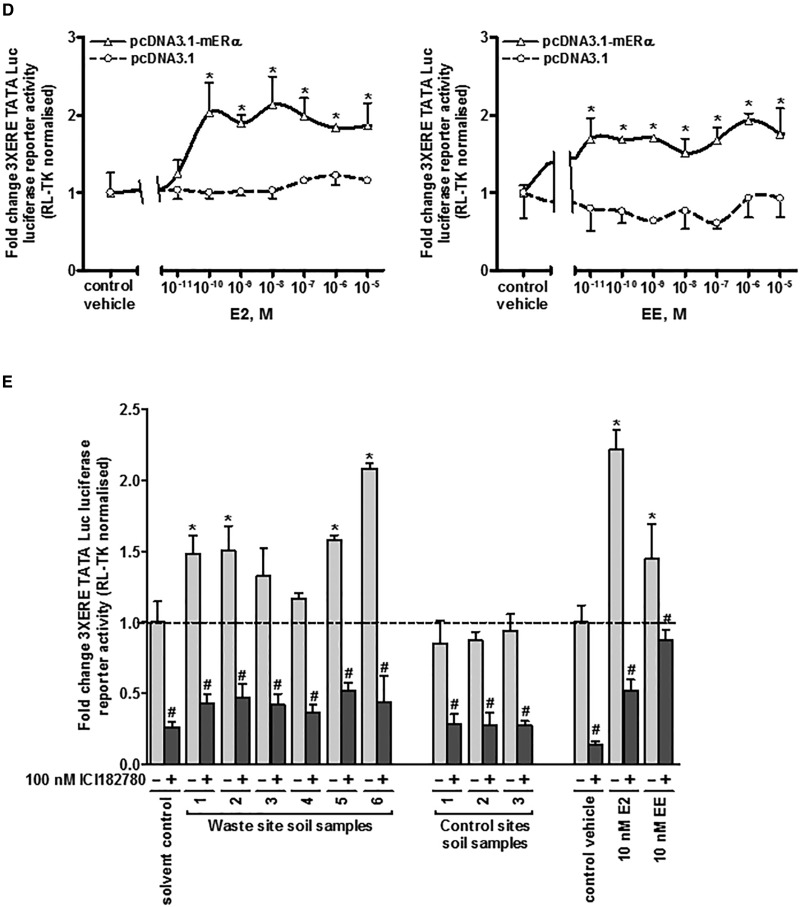
Extracts of soil samples in close proximity to a landfill site contain a chemical(s) that activate the mouse ERα in a murine ductal (LTPA) cell line. **A**, Western Blot (20 μg total protein/lane) for the expression of the mouse ERα protein in LTPA and HEK293 cells transfected with the expression construct encoding the mouse ERα cDNA sequence. **B**, immunocytochemical analysis for ERα expression in LTPA or HEK293 cells transfected with an expression vector encoding the mERα cDNA sequence. **C**, luciferase reporter gene (3XERE-TATA) assay in LTPA cells co-transfected with expression constructs encoding the indicated protein. Data are the mean and SD normalized luciferase activity from 3 entirely separate determinations of the same conditions performed within the same experiment, typical of at least 3 separate experiments performed at different times. *Significantly different (*P* < .05) versus the equivalent expression vector transfected DMSO vehicle-treated cells; #significantly different (*P* < .05) versus equivalent treatments in the absence of ICI182780-treated cells using the Student’s *t*-test (2-tailed). **D**, luciferase reporter gene (3XERE-TATA) assay in LTPA cells co-transfected with expression construct encoding the mERα and comparison to cells transfected with an empty expression construct. Data are the mean and SD normalized luciferase activity from 3 entirely separate determinations of the same conditions performed within the same experiment, typical of at least 3 separate experiments performed at different times. *Significantly different (*P* < .05) versus empty vector transfected cells treated with the same concentration of E2 or EE. **E**, luciferase reporter gene (3xERE-TATA) assay in LTPA cells. Cells were transfected as outlined in the methods section and treated with 0.1% v/v ethanol extracts, E2 or EE for 24 h prior to analysis. Data are the mean and SD normalized luciferase activity from 3 entirely separate determinations of the same conditions performed within the same experiment, typical of at least 3 separate experiments performed at different times. *Significantly different (*P* < .05) versus the equivalent expression vector transfected DMSO vehicle-treated cells; #significantly different (*P* < .05) versus equivalent treatments in the absence of ICI182780-treated cells using the Student’s *t*-test (2-tailed).

The mERα cDNA was cloned as outlined in the methods section and expressed in LTPA cells (603B cells were resistant to ERα expression, data not included). Figures 3A and B demonstrates low but detectable expression of mERα protein in these cells (compared with mouse uterus or to the mERα transfected HEK293 cell line or to high hERα-expressing MCF7 human breast cancer cells). LTPA cells expressing mERα trans-activated the 3XERE TATA Luc reporter gene constitutively (likely due to oestrogens present in the cell media) since the ER antagonist ICI182780 significantly reduced constitutive luciferase gene expression (Figure 3C). However, addition of 10nM E2 significantly further induced luciferase gene expression confirming that the mERα construct produced functional protein and that the LTPA cells express co-factors permissive for mERα transcriptional responsiveness. The response of the 3XERE TATA Luc reporter gene to E2 or EE was concentration-dependent (Figure 3D) and treating LTPA cells with extracts from soils demonstrated that the extracts which activated the hERα in human MCF7 cells also activated the mERα in LTPA cells, an effect that was blocked by the ER antagonist ICI182780 (Figure 3E).

**FIG. 4:**
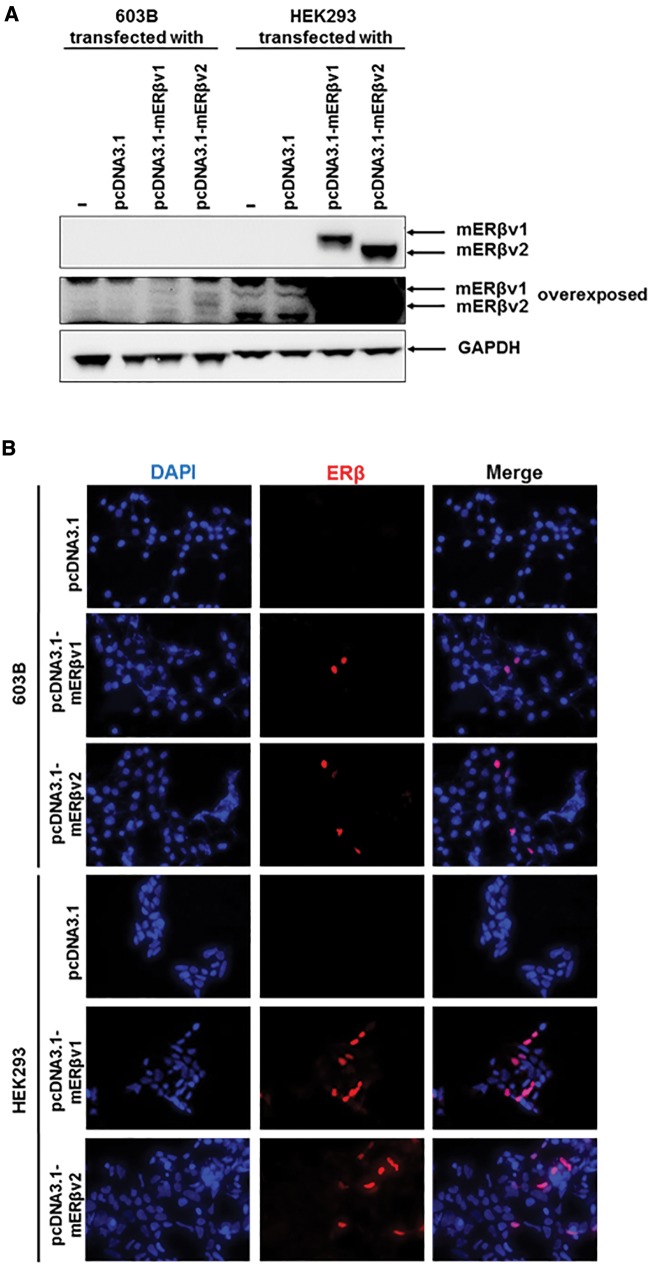

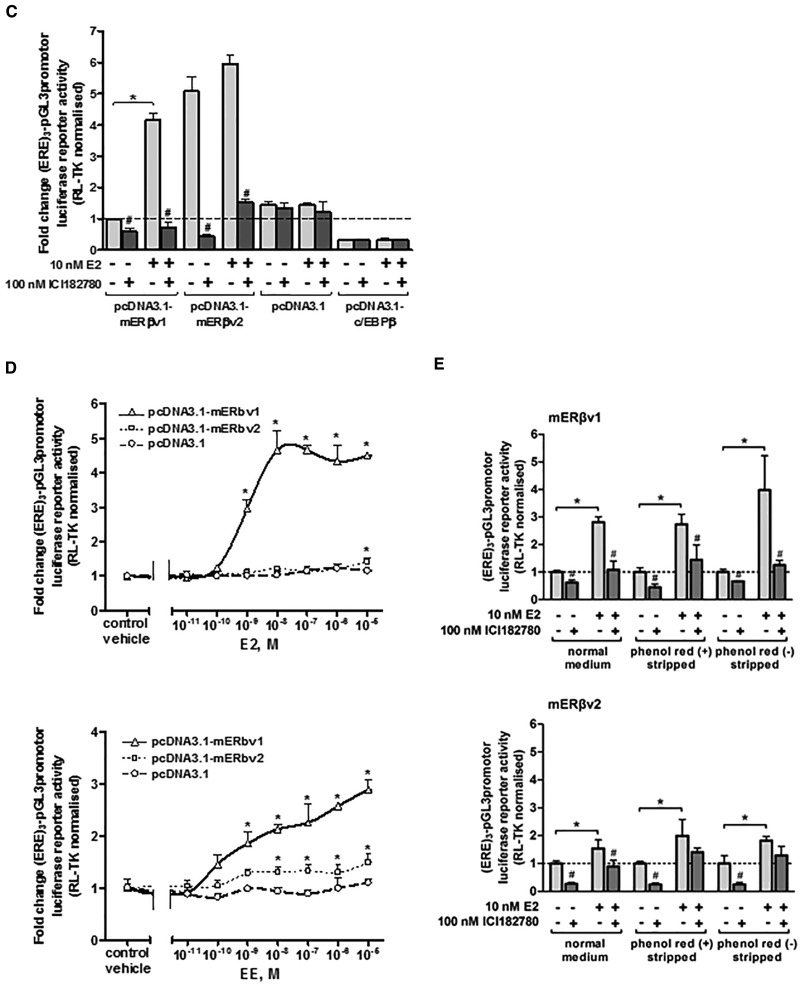

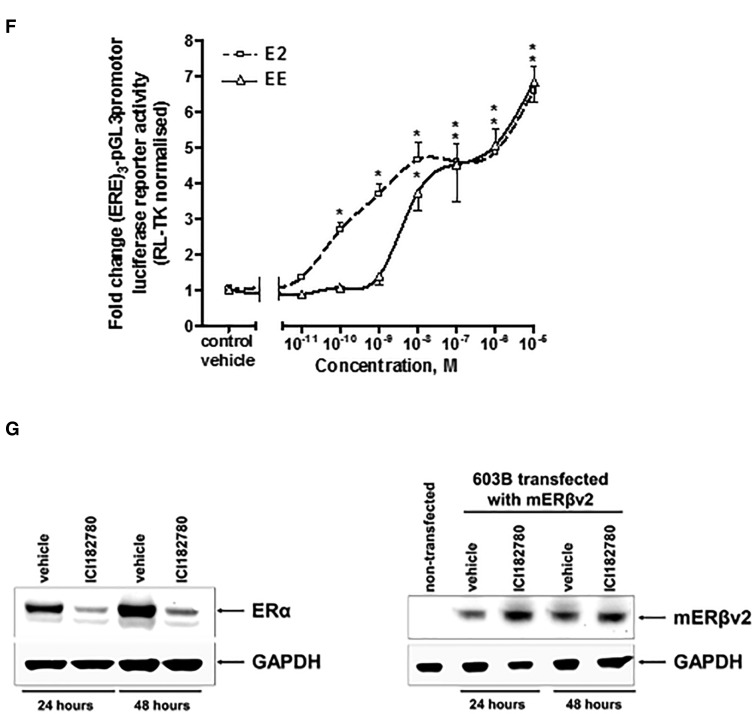
Extracts of soil samples in close proximity to a landfill site contain a chemical(s) that activate the mouse ERβ variants 1 and 2 in a murine pancreatohepatobiliary ductal (603B) cell line. **A**, Western Blot (20 μg total protein/lane) for the expression of the mouse ERβ proteins in LTPA and HEK293 cells transfected with the expression construct encoding either the mouse ERβ variant 1 (ERβv1) or ERβ variant 2 (ERβv2) cDNA sequences—theoretical molecular weights, variant 1, 63.23 kDa; variant 2, 61.21kDa). **B**, Immunocytochemical analysis for ERβ expression in 603B or HEK293 cells transfected with an expression vector encoding either the mERβ variant 1 or mERβ variant 2 cDNA sequence. **C**, luciferase reporter gene (ERE)_3_-pGL3promoter) assay in 603B cells co-transfected with expression constructs encoding the indicated protein. Data are the mean and SD normalized luciferase activity from 3 entirely separate determinations of the same conditions performed within the same experiment, typical of at least 3 separate experiments performed at different times. *Significantly different (*P* < .05) versus the equivalent expression vector transfected DMSO vehicle-treated cells; #significantly different (*P* < 0.05) versus equivalent treatments in the absence of ICI182780-treated cells using the Student’s *t*-test (2-tailed). **D**, luciferase reporter gene (ERE)_3_-pGL3promoter assay in 603B cells co-transfected with expression construct encoding the mERβv1 or mERβv2 and comparison to cells transfected with an empty expression construct. Cells were treated with the indicated concentration of either E2 (upper) or EE (lower). Data are the mean and SD normalized luciferase activity from 3 entirely separate determinations of the same conditions performed within the same experiment, typical of at least 3 separate experiments performed at different times. *Significantly different (*P* < 0.05) versus empty vector transfected cells treated with the same concentration of E2 or EE. **E**, luciferase reporter gene (ERE)_3_-pGL3promoter assay in 603B cells and co-transfected with the indicated expression construct (with RL-TK)—effect of performing the study in medium supplemented with normal serum, charcoal-stripped serum or charcoal-stripped serum and medium lacking phenol red. Cells were grown in the indicated media for 24 h before transfection. Twenty-four hours after transfection, cells were pre-treated with 100 nM ICI182780 or DMSO vehicle as indicated for 6 h followed by treatment with E2 or DMSO vehicle (± ICI182780) for 24 h. Data are the mean and SD normalized luciferase activity from 3 entirely separate determinations of the same conditions performed within the same experiment, typical of at least 3 separate experiments performed at different times. *Significantly different (*P* < .05) versus vehicle-treated cells grown in the equivalent medium using the Student’s *t*-test (2-tailed). ^#^Significantly different versus equivalent treatments in the absence of ICI182780 using the Student’s *t* test (2-tailed). **F**, luciferase reporter gene (ERE)_3_-pGL3promoter assay in 603B cells co-transfected with expression construct encoding the mERβv2 and pre-treated where indicated with 100 nM ICI182780 prior to addition of the indicated concentration of E2 or EE. Data are the mean and SD normalized luciferase activity from 3 entirely separate determinations of the same conditions performed within the same experiment, typical of at least 3 separate experiments performed at different times. *Significantly different (*P* < .05) versus empty vector transfected cells treated with the same concentration of E2. **G**, Western blot for the expression of the ERα in MCF-7 cells (left) and mERβv2 in 603B cells transfected with the pcDNA3.1-mERβv2 expression construct (right) after treatment with 100 nM ICI182780 or DMSO vehicle for 24 and 48 h (20 μg total protein/lane).

Two mERβ sequences were amplified by RT-PCR from mouse ovary RNA template (see Figure 2A) and after cloning and sequence analysis, both were observed to be identical to NM_207707.1 [full length; variant 1] and NM_010157.3 [variant 2] lacking an in-frame exon in the coding region, compared with mERβv1 resulting in a protein lacking amino acids 383–400 seen with mERβv1. For alignment of cDNA and amino acid sequences see Supplementary Figure S1. Figures 4A and B demonstrate that proteins of the predicted size were generated and localized to the nucleus in cells respectively when the proteins were expressed in HEK293 cells with low levels detectable in 603B cells. Figure 4C demonstrates that 603B cells expressing mERβv1 trans-activated the (ERE)_3_-pGL3promoter reporter gene constitutively (likely due to oestrogens present in the cell media) since co-addition of the ER antagonist ICI182780 significantly reduced luciferase gene expression. However, addition of 10 nM E2 significantly further induced luciferase gene expression confirming that the mERβv1 construct produced functional protein and that the 603B cells expressed co-factors permissive for mERβ responsiveness. In contrast, mERβv2 appeared to be constitutively fully activated since the ER antagonist ICI182780 significantly reduced luciferase gene expression to levels also seen with ICI182780 treatment with mERβv1 and addition of 10 nM E2 did not result in further induced luciferase gene expression (Figure 4C). Figure 4D demonstrates that the response of the (ERE)_3_-pGL3promoter reporter gene to E2 or EE was concentration-dependent when the cells expressed mERβv1. An absence or a limited response was observed with E2, and only a weak concentration-dependent response was observed with EE when mERβv2 was expressed (Figs. 4D and E).

Serum constituents and the culture medium pH indicator phenol red have been shown to be activators of ERs (Berthois *et al.*, 1986). We therefore examined the influence of medium constituents and phenol red on mERβv1 and ERβv2 transcriptional functional to determine whether ERβv2 is more readily activated by culture medium constituents. Figure 4E suggests that the removal of these potential endogenous culture media constituents has little overall effect on fold activation by E2 or fold inhibition by ICI182780. Notably, the apparent inhibition of constitutive mERβv2 transcriptional function by ICI182780 is not lost when the assay is performed in cells cultured in charcoal-stripped and phenol red-free medium, suggesting these factors were not responsible for its constitutive activation. Figure 4F indicates however, that de-activation of the mERβv2 in 603B cells with pre-treatment with IC182780 followed by a washout and treatment with a range of E2 or EE concentrations results in a robust dose–response increase in reporter gene expression.

Previous work in mammary cells and related cell lines has shown that the human ERα is subject to both an antagonist effect by ICI182780, but also to a mechanistically-distinct 26S proteasomal (MG132-inhibitable) mediated degradation of the protein (Wardell *et al.*, 2011). These observations have given rise to the term “selective estrogen receptor degrader (SERD)” for ICI182780. Thus, ligand-induced degradation of ERα by ICI182780 can be saturated through adenoviral-mediated ERα over-expression, but its ICI182780 antagonism of ERα is retained (Wardell *et al.*, 2011). In contrast to the human ERα, SERD activity for ICI182780 is not observed for the human ERβ and in MCF7 cells, ICI182780 actually stabilized ERβ protein (Peekhaus *et al.*, 2004). Figure 4G demonstrates that in our hands, ICI182780 treatment resulted in a loss of hERα protein in MCF7 cells (in agreement with Wardell *et al.*, 2011), but not mERβv2 in 603B cells. The lack of any effect of ICI182780 on mERβv2 protein in 603B cells was therefore similar to the effects observed on hERβ by Peekhaus *et al.* (2004) and suggests that re-activation of mERβv2 by ICI182780 in 603B cells was not associated with SERD-like changes in protein stability.

Therefore, the murine mERβv2 was constitutively active and required de-activation to be responsive to oestrogens E2 or EE in 603B cells.

**FIG. 5:**
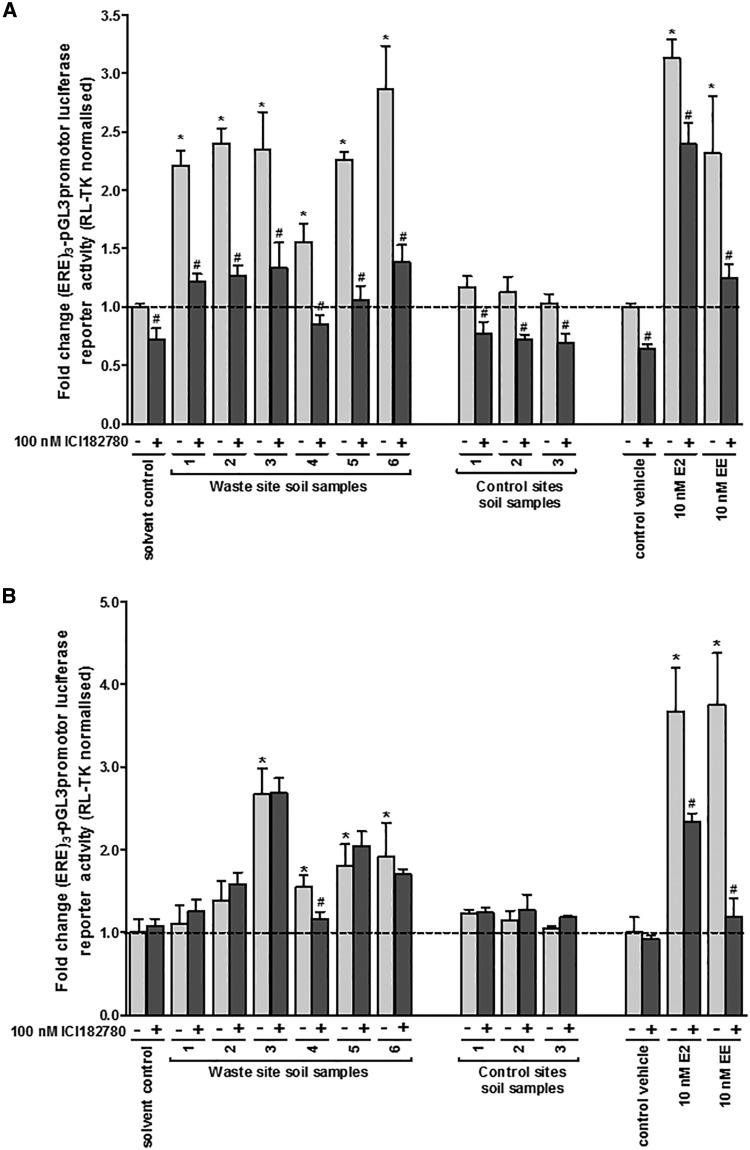
The mERβv2 is activated by landfill site soil xtracts after ICI182780 treatment and washout, but resists subsequent antagonism by ICI182780 in contrast to E2 and EE. **A**, luciferase reporter gene (ERE)_3_-pGL3promoter assay in 603B cells. Cells were transfected with mERβv1 as outlined in the methods section treated with 0.1% v/v ethanol extracts, E2 or EE for 24 h prior to analysis. Data are the mean and SD normalized luciferase activity from 3 entirely separate determinations of the same conditions performed within the same experiment, typical of at least 3 separate experiments performed at different times. *Significantly different (*P* < .05) versus the equivalent expression vector transfected DMSO vehicle-treated cells; #significantly different (*P* < .05) versus equivalent treatments in the absence of ICI182780-treated cells using the Student’s *t* test (2-tailed). **B**, luciferase reporter gene (ERE)_3_-pGL3promoter assay in 603B cells. Cells were transfected with mERβv2 as outlined in the “Methods” section and, after 24 h, pre-treated with ICI182780 for 6 h to de-activate constitutively active mERβv2. Cells were then washed with sterile PBS to reduce the level of ER antagonist to negligible levels followed by treatment with 0.1% v/v ethanol extracts, oestradiol (E2) or ethinylestradiol (EE) in the absence or presence of ICI182780 for 24 h prior to analysis. Data are the mean and SD normalized luciferase activity from 3 entirely separate determinations of the same conditions performed within the same experiment, typical of at least 3 separate experiments performed at different times. *Significantly different (*P* < .05) versus the equivalent expression vector transfected DMSO vehicle-treated cells; #significantly different (*P* < .05) versus equivalent treatments in the absence of ICI182780-treated cells using the Student’s *t* test (2-tailed).

Treating 603B cells with extracts from soils demonstrated that the extracts which activated the hERα in MCF7 cells also activated the mERβv1in 603B cells (Figure 5A). Prior deactivation with ICI182780 also permitted the mERβv2 to be activated by some of the extracts from soils. In some cases, such as soil sample 1, only mERβv1 was activated whereas soil sample 3 contained a chemical(s) that activated both of the mERβv1 and mERβv2 (Figure 5B). However, despite effective antagonism by ICI182780 versus E2- or EE-activation of mERβv2, the majority of soil extract samples were resistant to subsequent antagonism by ICI182780 (Figure 5B).

#### ER Expression in Mouse Cholangiocytes

**FIG. 6:**
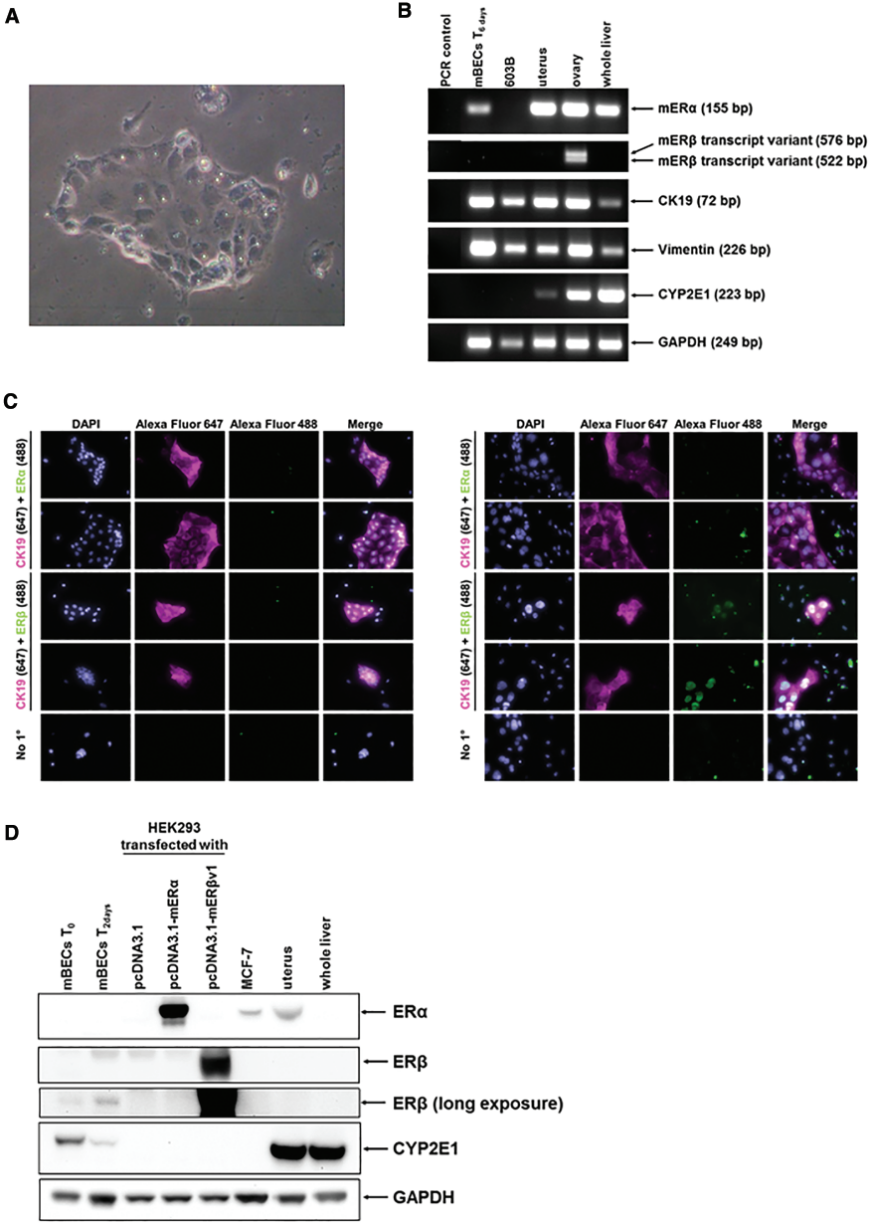
Murine cholangiocytes express ERβ at isolation and early culture periods. **A**, light photomicrograph of cholangiocytes in culture for 24 h. **B**, RT-PCR analysis for the indicated mRNA transcript in mouse cholangiocytes at isolation, 603B cells or the indicated murine tissue. **C**, immunocytochemical analysis for ER expression in cholangiocytes. Left panels after 1 day of culture, right panels after 4 days of culture. **D**, Western blot for the indicated protein in primary mouse cholangiocytes, cell lines or mouse tissues as indicated (20 μg total protein/lane).

Although ERα and ERβ are known to be expressed in human and rat cholangiocytes and markedly upregulated in disease settings (Alvaro *et al.*, 2004, 2006), there is limited data in the literature regarding mER expression in mouse cholangiocytes. Xia *et al.* (2012) report that both mERα and mERβ mRNA transcripts are expressed in mouse cholangiocytes and that expression of both increase with culture. Isse *et al.* (2010) demonstrated that the role of ERα in murine and human cholangiocytes—at least in females—is in the regulation of interleukin 6 expression. Cholangiocytes were therefore isolated from mouse liver as outlined in the methods section, giving rise to a population of cells that were morphologically pure at isolation and which proliferated in culture (Figure 6A). The cells were expandable over 1–2 passages but then either senesced or became overgrown by fibroblasts and/or underwent epithelial-mesenchymal trans-differentiation to fibroblasts as evidenced by high vimentin mRNA expression (Figure 6B). In our hands, there was little evidence for expression of mER proteins in isolated mouse cholangiocytes. However, with culture, during which time the cells expanded in number and retained their epithelial morphology, the cells expressed detectable levels of mERβ as determined by immunocytochemistry (Figure 6C) and Western blotting (Figure 6D).

#### Acute Exposure to Oestrogen Positive Soil Extract Results in Cholangiopathic Injury in the Absence of any Hepatocellular Injury

**FIG. 7:**
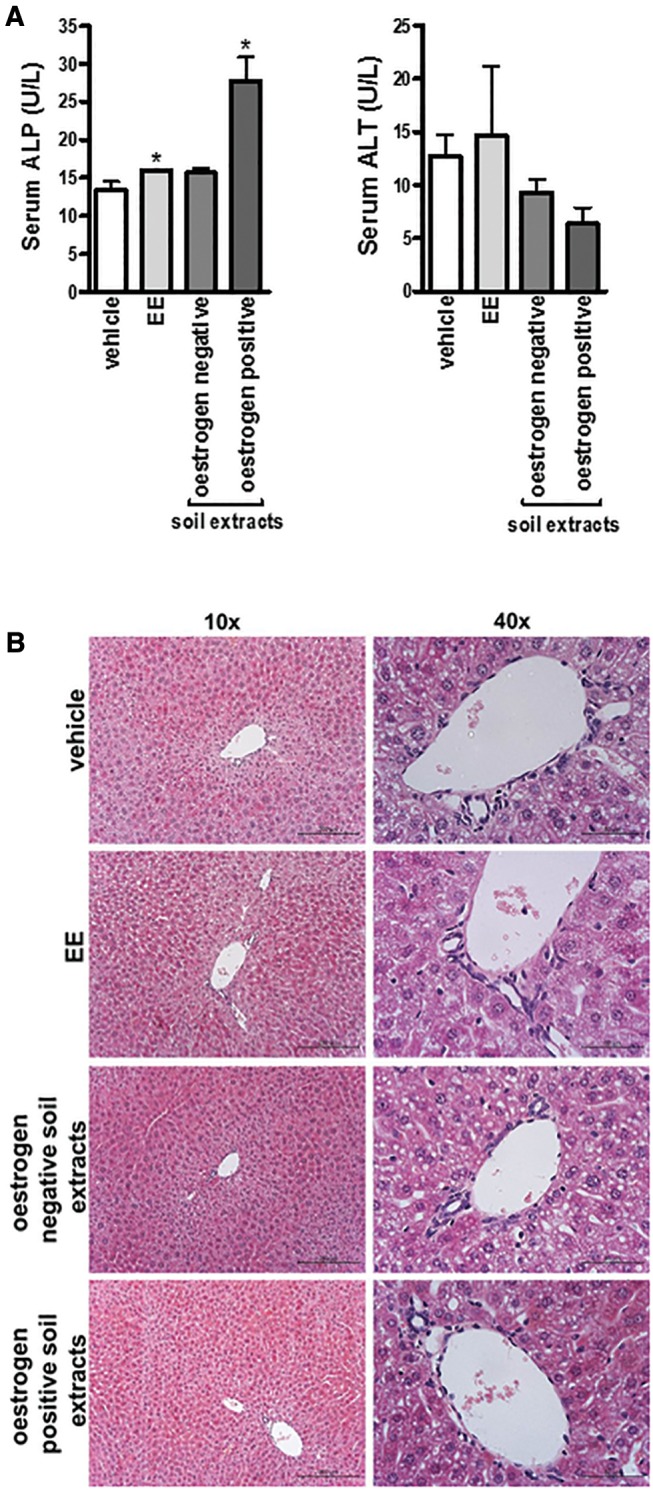
Acute exposure to soil extracts containing xenoestrogens results in a cholestatic injury in the absence of any overt pathology. **A**, Serum ALP (left) and ALT (right). Data are the mean and SD activities for the indicated group 24 h after exposure as outlined in the “Methods” section, *Significantly different (*P* < 0.05) versus vehicle control treated mice. **B**, Haematoxylin and eosin stained liver sections from the indicated group, typical views shown with right panels, magnified view of the periportal regions.

To examine whether any of the soil extracts have any hepatic effects, soil extracts were combined and concentrated and male mice were acutely exposed to extracts via an intraperitoneal route of exposure. Figure 7A demonstrates that the combined soil extracts demonstrating oestrogenicity resulted in a significant increase in serum alkaline phosphatase (ALP) activity, a clinical marker for cholangiopathy, although there was a significant decrease in serum alanine aminotransferase activity (ALT), a marker for hepatocellular injury. EE treatment also resulted in a small but significant increase in ALP and in some mice, elevated serum ALT, although overall there was not a significant increase over this period of exposure. However, no marked histological changes were noted in liver sections (Figure 7B), suggesting that these changes in clinical chemical markers occurred in the absence of any overt cell death.

## DISCUSSION

The data in this article provide proof of concept that it is possible to detect xenoestrogenic activity in soil extracts and that measurably high levels can be detected in landfill site soil samples compared with control site soil samples not in proximity to landfill sites and not expected to be contaminated with chemicals such as pesticides. The study has been performed in an historic mining area therefore, to gain access to the most recently deposited material, surface sediments were taken. To our knowledge, this approach has not been employed before. The xenoestrogens present in landfill site soils were shown to activate the hERα in a dose–response manner, an effect that was antagonized by the ER antagonist ICI182780. However, the chemical identity(ies) of the xenoestrogen(s) present in the soil around the landfill site were not investigated. Given the wide range of natural and man-made chemicals shown to activate ERs (Axon *et al.*, 2012; Kuiper *et al.*, 1998; Laws *et al.*, 2000) and the variety of chemicals potentially present in soils around a landfill site, the responses observed are likely to be related to mixtures of agonistic and antagonistic chemicals in combination with non-interacting ER chemicals. Therefore, identification of the xenoestrogen(s) responsible for oestrogenic activities is a significant task. However, by estimating the oestrogenic activity of cell extracts, it is possible to estimate the biological agonistic activity of the chemical mixture and to determine an “E2 equivalent” activity. By applying this principle, the approximate EC_50%_ for landfill site 3 (1/5000 dilution) and E2 activation (10^−^^12^ M) of the hERα, these data suggest that the concentration of xenoestrogens in landfill site soil 3 was approximately 5 nM E2 equivalents (or likely within the range of 1–10 nM E2 equivalents).

In order to be in a position to test the potential toxicological effects of xenoestrogen in man, an animal model is required to take into account exposure routes, toxicokinetic and toxicodynamic effects. Given the abundance of transgenic variants available, the mouse as a species was selected for its potential in future mechanistic in vivo studies. Given the focus of this study on the liver and the known expression and function of ERα in hepatocytes, the hepatocyte would have been the preferred option for screening the oestrogenicity of chemicals (and remains a key goal in future studies). However, since hepatocytes do not undergo mitosis to any significant extent in vitro, transfection efficiencies with hepatocytes are often too low. To generate high signal to noise readouts that are appropriately controlled, and given the need to screen a large set of samples with appropriate replication (ie, at semi-high throughput), the use of mouse hepatocytes in these studies was not a realistic option. Given the apparent role of ERs in human cholangiocyte biology, pancreatohepatobiliary ductal cell lines were selected for screening studies. The availability of pancreatohepatobiliary ductal cell lines in general is limited and in our hands, mERα was not functionally efficiently activated by any oestrogen in the cholangiocyte 603B cells, only in the pancreatic ductal LTPA cell line. The converse was the case for mERβ variants. The reason(s) for this is not clear but may be associated with a divergence between these cell types and the presence of factors required for mER transcriptional function. There may also be limited comparability between human and murine cholangiocyte ER expression which may impact on the relevance of observations in mice for man. However, should this difference in murine ERα and ERβ expression extend to human pancreatohepatobiliary tissues, it would suggest that hERβ transcriptional function predominates in cholangiocytes (when expressed, such as in disease settings). The data from these current studies indicate however, that the landfill site soil extracts containing xenoestrogen(s) activate the mERα with similar potency to the hERα and therefore the mouse may be a good model for examining potential endocrine disruptor properties of these extracts *in vivo* (eg, using the rodent uterotrophic bioassay).

In terms of the ERβ, the mouse has 2 variant transcripts which generate distinct mERβ proteins. Both transcripts were present in mouse ovary. The mERβv1 protein was transcriptionally activated by landfill site soil extracts containing xenoestrogen(s) with similar potency to the hERα and mERα. However, the mERβv2 protein was near constitutively transcriptionally fully activated when expressed in 603B cells and required inactivation by pre-treatment with ICI18270 and wash removal for dose–response activation by E2 or EE. To our knowledge, this has not been previously described, and may be a unique feature of the context in which the receptor is expressed (ie, in cholangiocytes). Furthermore, of potential significant toxicological significance, this receptor retains its sensitivity to antagonism by ICI182780 when activated by E2 or EE, but not by landfill site soil-based xenoestrogens. Assuming that the xenoeostrogen(s) interacts similarly with the ligand binding region of the mERβv2, this observation suggests that the(ir) rate of dissociation is significantly reduced compared with E2. Alternatively, the xenoestrogen(s) may interact and activate the receptor via a different mechanism (such as through a different binding site), rendering the receptor insensitive to ICI182780 antagonism.

Multiple variant hERβ variant proteins generated from the hERβ gene have been described in man (Green *et al.*, 2008). Interrogation of the NCBI database at time of submission for the existence of variant hERβ transcripts indicates that at least 7 transcript variants have thus far been identified, leading to 5 variant proteins. Clustal alignment of the human and murine ERβ proteins (Supplementary Figure S2) indicates that all hERβ proteins lack the 18 amino acid insertion present in the mouse ERβ variant 1 protein and therefore hERβ proteins are more likely to respond to oestrogens and xenoestrogens similarly to the mERβv2 protein.

The functional role of ERβ in cholangiocytes under normal conditions is likely to be negligible since its expression is low. However, in clinical disease situations, it has been reported that ERβ expression is markedly up-regulated (Alvaro *et al.*, 2006) and that likely, ERβ antagonizes the pro-proliferative effects of hERα in man (Alvaro *et al.*, 2006). Thus, an ERβ-selective agonist induced apoptosis in cholangiocarcinoma (Marzioni *et al.*, 2012). Thus, it is tempting to propose that the role—in part—xenoestrogens could play in the development of PBC is via an inhibition of bile duct proliferation and/or promotion of vanishing bile duct syndrome through a sustained activation of ERβ in cholangiocytes after an initial cholestatic insult (be it ER dependent or independent, see Supplementary Figure S3 for immunohistochemistry for ER expression in mouse liver; see Supplementary Figure S4 for schematic diagram).

PBC is thought to be triggered in genetically pre-disposed individuals through exposure to an unknown chemical(s) and/or infectious agent(s). There is an association between use of hormone replacement therapies and increased risk of having PBC (Gershwin *et al*., 2005). However, it would seem unlikely that an endocrine disruptor such as an ERβ agonist alone could be the trigger for such a disease, on the basis of exposure alone. Taking into consideration the mass of soil collected, the extraction procedure and the final volume of the ethanol, the concentration of E2 equivalents in landfill site soil sample 3 is calculated to be 1200 pmol/kg soil (or in the range 240–2400 pmol E2 equivalents/kg soil). If the oestrogenic activity in landfill site soil 3 was associated solely with E2, this is equivalent to 326 ng E2/kg soil (or in the range 65.2–652 ng E2 equivalents/kg soil). Typical levels of E2 in a sexually mature adult female are in the range of 200–1000 pM in serum (Sherman and Korenman, 1975; Rinaldi *et al.*, 2006). Assuming a serum volume of 4 l and that only serum levels of E2 are relevant, this suggests total body E2 levels of 800–4000 pmol/person. Assuming complete oral absorption and similar distribution, metabolism and excretion to E2, to achieve biologically equivalent levels of oestrogenic activity from landfill site soil 3 xenoestrogen would require exposure to 1.5 kg soil/person (or in the range 3.3–0.33 kg soil/person) to lead to low level E2 activity and 3.3 kg soil/person (or in the range [16.7–1.67 kg soil/person] to achieve high E2 activity. Given these considerations, it is unlikely that soil-based xenoestrogen exposure alone could trigger a PBC-like response, but rather there may be the possibility that it is a component of a trigger and/or sustaining process. Such a trigger process might also include a cholestatic injury, such as exposure to chemicals that mimic lipoic acid, resulting in their incorporation into mitochondrial proteins and which accounts for anti-mitochondrial antibodies associated with the disease.

## SUPPLEMENTARY DATA

Supplementary data are available at *Toxicological Sciences* online.

## FUNDING

This work was funded by a grant from the Institute of Sustainability (Newcastle University) and was in part, funded by the National Institute for Health Research Health Protection Research Unit (NIHR HPRU) in Chemical and Radiation Threats and Hazards in partnership with Public Health England (PHE) and the MRC (in the form of an ITTP studentship to A.C.L.). NIHR HPRU funding was used for the part of the research that did not involve any live animals. The views expressed are those of the author(s) and not necessarily those of the NHS, the NIHR, the Department of Health or Public Health England.

## Supplementary Material

Supplementary Data
