# Supplementary material for: Environmental Xenoestrogens Super-Activate a Variant Murine ER Beta in Cholangiocytes
Source: Toxicol Sci. 2016 Dec 24;156(1):54–71. doi: 10.1093/toxsci/kfw234 (PMC5356623; doi:10.1093/toxsci/kfw234)
Supplement: Supplementary Data [file kfw234_Supp.zip › toxsci-16-0476-File012.pdf]

```
CLUSTAL O(1.2.1) multiple sequence alignment


ERb_v1          ATGTCCATCTGTGCCTCTTCTCACAAGGATTTTTCTCAGCTGAGACCTACGCAAGACATG
ERb_v2          ATGTCCATCTGTGCCTCTTCTCACAAGGATTTTTCTCAGCTGAGACCTACGCAAGACATG
                ************************************************************

ERb_v1          GAGATCAAAAACTCACCATCAAGCCTTACTTCCCCTGCTTCTTATAACTGTAGCCAGTCC
ERb_v2          GAGATCAAAAACTCACCATCAAGCCTTACTTCCCCTGCTTCTTATAACTGTAGCCAGTCC
                ************************************************************

ERb_v1          ATCCTACCCTTGGAGCATGGTCCCATCTATATCCCTTCCTCCTATGTAGAGAGCCGTCAC
ERb_v2          ATCCTACCCTTGGAGCATGGTCCCATCTATATCCCTTCCTCCTATGTAGAGAGCCGTCAC
                ************************************************************

ERb_v1          GAATACTCAGCCATGACATTCTACAGTCCTGCTGTGATGAACTACAGTGTTCCCAGCAGC
ERb_v2          GAATACTCAGCCATGACATTCTACAGTCCTGCTGTGATGAACTACAGTGTTCCCAGCAGC
                ************************************************************

ERb_v1          ACCGGTAACCTGGAAGGTGGGCCTGTTCGCCAGACTGCAAGCCCAAATGTGCTATGGCCA
ERb_v2          ACCGGTAACCTGGAAGGTGGGCCTGTTCGCCAGACTGCAAGCCCAAATGTGCTATGGCCA
                ************************************************************

ERb_v1          ACTTCTGGACACCTCTCTCCTTTAGCCACCCACTGCCAATCATCGCTTCTCTATGCAGAA
ERb_v2          ACTTCTGGACACCTCTCTCCTTTAGCCACCCACTGCCAATCATCGCTTCTCTATGCAGAA
                ************************************************************

ERb_v1          CCTCAAAAGAGTCCTTGGTGTGAAGCAAGATCACTAGAACACACCTTGCCTGTAAACAGA
ERb_v2          CCTCAAAAGAGTCCTTGGTGTGAAGCAAGATCACTAGAACACACCTTGCCTGTAAACAGA
                ************************************************************

ERb_v1          GAGACCCTGAAGAGGAAGCTTGGCGGGAGCGGTTGTGCCAGCCCTGTTACTAGTCCAAGC
ERb_v2          GAGACCCTGAAGAGGAAGCTTGGCGGGAGCGGTTGTGCCAGCCCTGTTACTAGTCCAAGC
                ************************************************************

ERb_v1          GCCAAGAGGGATGCTCACTTCTGCGCCGTCTGCAGTGATTATGCATCTGGGTATCATTAC
ERb_v2          GCCAAGAGGGATGCTCACTTCTGCGCCGTCTGCAGTGATTATGCATCTGGGTATCATTAC
                ************************************************************

ERb_v1          GGTGTCTGGTCCTGTGAAGGATGTAAGGCCTTTTTTAAAAGAAGCATTCAAGGACATAAT
ERb_v2          GGTGTCTGGTCCTGTGAAGGATGTAAGGCCTTTTTTAAAAGAAGCATTCAAGGACATAAT
                ************************************************************

ERb_v1          GACTATATCTGTCCAGCCACGAATCAGTGTACCATAGACAAGAACCGGCGTAAAAGCTGC
ERb_v2          GACTATATCTGTCCAGCCACGAATCAGTGTACCATAGACAAGAACCGGCGTAAAAGCTGC
                ************************************************************

ERb_v1          CAGGCCTGCCGACTTCGCAAGTGTTACGAAGTAGGAATGGTCAAGTGTGGATCCAGGAGA
ERb_v2          CAGGCCTGCCGACTTCGCAAGTGTTACGAAGTAGGAATGGTCAAGTGTGGATCCAGGAGA
                ************************************************************

ERb_v1          GAAAGGTGTGGGTACCGAATAGTACGAAGACAGAGAAGTGCCAGCGAGCAGGTGCATTGC
ERb_v2          GAAAGGTGTGGGTACCGAATAGTACGAAGACAGAGAAGTGCCAGCGAGCAGGTGCATTGC
                ************************************************************

ERb_v1          CTGAACAAAGCCAAGAGAACCAGTGGGCACACACCCCGGGTGAAGGAGCTACTGCTGAAC
ERb_v2          CTGAACAAAGCCAAGAGAACCAGTGGGCACACACCCCGGGTGAAGGAGCTACTGCTGAAC
                ************************************************************

ERb_v1          TCTCTGAGTCCCGAGCAGCTGGTGCTCACCCTGCTGGAAGCTGAGCCACCCAATGTGCTA
ERb_v2          TCTCTGAGTCCCGAGCAGCTGGTGCTCACCCTGCTGGAAGCTGAGCCACCCAATGTGCTA
                ************************************************************

ERb_v1          GTGAGCCGTCCCAGCATGCCCTTCACCGAGGCCTCCATGATGATGTCCCTCACGAAGCTG
ERb_v2          GTGAGCCGTCCCAGCATGCCCTTCACCGAGGCCTCCATGATGATGTCCCTCACGAAGCTG
                ************************************************************
```

```
ERb_v1       GCTGACAAGGAACTGGTGCACATGATTGGCTGGGCCAAGAAAATCCCTGGCTTTGTGGAG
ERb_v2       GCTGACAAGGAACTGGTGCACATGATTGGCTGGGCCAAGAAAATCCCTGGCTTTGTGGAG
             ************************************************************

ERb_v1       CTCAGCCTGTTGGACCAAGTCCGCCTCTTGGAAAGCTGCTGGATGGAGGTGCTGATGGTG
ERb_v2       CTCAGCCTGTTGGACCAAGTCCGCCTCTTGGAAAGCTGCTGGATGGAGGTGCTGATGGTG
             ************************************************************

ERb_v1       GGGCTGATGTGGCGCTCCATCGACCACCCCGGCAAGCTCATCTTTGCTCCAGACCTCGTT
ERb_v2       GGGCTGATGTGGCGCTCCATCGACCACCCCGGCAAGCTCATCTTTGCTCCAGACCTCGTT
             ************************************************************

ERb_v1       CTGGACAGGTCCTCAGAAGACCCTCACTGGCACGTTGCGCAGACGAAGAGTGCTGTCCCA
                     R  S  S  E  D  P  H  W  H  V  A  Q  T  K  S  A  V  P

ERb_v2       CTGGACA-----------------------------------------------------
             *******

ERb_v1       AGGGATGAGGGGAAGTGCGTGGAAGGGATTCTGGAAATCTTTGACATGCTCCTGGCGACG
ERb_v2       -GGGATGAGGGGAAGTGCGTGGAAGGGATTCTGGAAATCTTTGACATGCTCCTGGCGACG
              ***********************************************************

ERb_v1       ACGGCACGGTTCCGTGAGTTAAAACTGCAGCACAAAGAATATCTGTGTGTGAAGGCCATG
ERb_v2       ACGGCACGGTTCCGTGAGTTAAAACTGCAGCACAAAGAATATCTGTGTGTGAAGGCCATG
             ************************************************************

ERb_v1       ATTCTCCTCAACTCCAGTATGTACCCCTTGGCTACCGCAAGCCAGGAAGCAGAGAGTAGC
ERb_v2       ATTCTCCTCAACTCCAGTATGTACCCCTTGGCTACCGCAAGCCAGGAAGCAGAGAGTAGC
             ************************************************************

ERb_v1       CGGAAGCTGACACACCTATTGAACGCAGTGACAGATGCCCTGGTCTGGGTGATTTCGAAG
ERb_v2       CGGAAGCTGACACACCTATTGAACGCAGTGACAGATGCCCTGGTCTGGGTGATTTCGAAG
             ************************************************************

ERb_v1       AGTGGAATCTCTTCCCAGCAGCAGTCAGTCCGTCTGGCCAACCTCCTGATGCTTCTTTCT
ERb_v2       AGTGGAATCTCTTCCCAGCAGCAGTCAGTCCGTCTGGCCAACCTCCTGATGCTTCTTTCT
             ************************************************************

ERb_v1       CATGTCAGGCACATCAGTAACAAGGGCATGGAACATCTGCTCAGCATGAAGTGCAAAAAT
ERb_v2       CATGTCAGGCACATCAGTAACAAGGGCATGGAACATCTGCTCAGCATGAAGTGCAAAAAT
             ************************************************************

ERb_v1       GTGGTCCCGGTGTACGACCTGCTGCTGGAGATGCTGAATGCTCACACGCTTCGAGGGTAC
ERb_v2       GTGGTCCCGGTGTACGACCTGCTGCTGGAGATGCTGAATGCTCACACGCTTCGAGGGTAC
             ************************************************************

ERb_v1       AAGTCCTCAATCTCGGGGTCTGAGTGCTGCTCGACAGAGGACAGTAAGAGCAAAGAGGGC
ERb_v2       AAGTCCTCAATCTCGGGGTCTGAGTGCTGCTCGACAGAGGACAGTAAGAGCAAAGAGGGC
             ************************************************************

ERb_v1       TCCCAGAACCTCCAGTCACAGTGA
ERb_v2       TCCCAGAACCTCCAGTCACAGTGA
             ************************
```
