# Supplementary material for: Environmental Xenoestrogens Super-Activate a Variant Murine ER Beta in Cholangiocytes
Source: Toxicol Sci. 2016 Dec 24;156(1):54–71. doi: 10.1093/toxsci/kfw234 (PMC5356623; doi:10.1093/toxsci/kfw234)
Supplement: Supplementary Data [file kfw234_Supp.zip › toxsci-16-0476-File013.pdf]

```
CLUSTAL O(1.2.2) multiple sequence alignment

mERb_v1      MSICASSHKDFSQLRPTQDMEIKNSPSSLTSPASYNCSQSILPLEHGPIYIPSSYVESRH
mERb_v2      MSICASSHKDFSQLRPTQDMEIKNSPSSLTSPASYNCSQSILPLEHGPIYIPSSYVESRH
hERb_v6      -------------------MDIKNSPSSLNSPSSYNCSQSILPLEHGSIYIPSSYVDSHH
hERb_v1      -------------------MDIKNSPSSLNSPSSYNCSQSILPLEHGSIYIPSSYVDSHH
hERb_v3      -------------------MDIKNSPSSLNSPSSYNCSQSILPLEHGSIYIPSSYVDSHH
hERb_v2      -------------------MDIKNSPSSLNSPSSYNCSQSILPLEHGSIYIPSSYVDSHH
hERb_5       -------------------MDIKNSPSSLNSPSSYNCSQSILPLEHGSIYIPSSYVDSHH
                                *:********.**:************** ********:*:*

mERb_v1      EYSAMTFYSPAVMNYSVPSSTGNLEGGPVRQTASPNVLWPTSGHLSPLATHCQSSLLYAE
mERb_v2      EYSAMTFYSPAVMNYSVPSSTGNLEGGPVRQTASPNVLWPTSGHLSPLATHCQSSLLYAE
hERb_v6      EYPAMTFYSPAVMNYSIPSNVTNLEGGPGRQTTSPNVLWPTPGHLSPLVVHRQLSHLYAE
hERb_v1      EYPAMTFYSPAVMNYSIPSNVTNLEGGPGRQTTSPNVLWPTPGHLSPLVVHRQLSHLYAE
hERb_v3      EYPAMTFYSPAVMNYSIPSNVTNLEGGPGRQTTSPNVLWPTPGHLSPLVVHRQLSHLYAE
hERb_v2      EYPAMTFYSPAVMNYSIPSNVTNLEGGPGRQTTSPNVLWPTPGHLSPLVVHRQLSHLYAE
hERb_5       EYPAMTFYSPAVMNYSIPSNVTNLEGGPGRQTTSPNVLWPTPGHLSPLVVHRQLSHLYAE
             ** *************:**.. ****** ***:******** ******..* * * ****

mERb_v1      PQKSPWCEARSLEHTLPVNRETLKRKLGGSGCASPVTSPSAKRDAHFCAVCSDYASGYHY
mERb_v2      PQKSPWCEARSLEHTLPVNRETLKRKLGGSGCASPVTSPSAKRDAHFCAVCSDYASGYHY
hERb_v6      PQKSPWCEARSLEHTLPVNRETLKRKVSGNRCASPVTGPGSKRDAHFCAVCSDYASGYHY
hERb_v1      PQKSPWCEARSLEHTLPVNRETLKRKVSGNRCASPVTGPGSKRDAHFCAVCSDYASGYHY
hERb_v3      PQKSPWCEARSLEHTLPVNRETLKRKVSGNRCASPVTGPGSKRDAHFCAVCSDYASGYHY
hERb_v2      PQKSPWCEARSLEHTLPVNRETLKRKVSGNRCASPVTGPGSKRDAHFCAVCSDYASGYHY
hERb_5       PQKSPWCEARSLEHTLPVNRETLKRKVSGNRCASPVTGPGSKRDAHFCAVCSDYASGYHY
             **************************:.*. ******.*.:*******************

mERb_v1      GVWSCEGCKAFFKRSIQGHNDYICPATNQCTIDKNRRKSCQACRLRKCYEVGMVKCGSRR
mERb_v2      GVWSCEGCKAFFKRSIQGHNDYICPATNQCTIDKNRRKSCQACRLRKCYEVGMVKCGSRR
hERb_v6      GVWSCEGCKAFFKRSIQGHNDYICPATNQCTIDKNRRKSCQACRLRKCYEVGMVKCGSRR
hERb_v1      GVWSCEGCKAFFKRSIQGHNDYICPATNQCTIDKNRRKSCQACRLRKCYEVGMVKCGSRR
hERb_v3      GVWSCEGCKAFFKRSIQGHNDYICPATNQCTIDKNRRKSCQACRLRKCYEVGMVKCGSRR
hERb_v2      GVWSCEGCKAFFKRSIQGHNDYICPATNQCTIDKNRRKSCQACRLRKCYEVGMVKCGSRR
hERb_5       GVWSCEGCKAFFKRSIQGHNDYICPATNQCTIDKNRRKSCQACRLRKCYEVGMVKCGSRR
             ************************************************************

mERb_v1      ERCGYRIVRRQRSASEQVHCLNKAKRTSGHTPRVKELLLNSLSPEQLVLTLLEAEPPNVL
mERb_v2      ERCGYRIVRRQRSASEQVHCLNKAKRTSGHTPRVKELLLNSLSPEQLVLTLLEAEPPNVL
hERb_v6      ERCGYRLVRRQRSADEQLHCAGKAKRSGGHAPRVRELLLDALSPEQLVLTLLEAEPPHVL
hERb_v1      ERCGYRLVRRQRSADEQLHCAGKAKRSGGHAPRVRELLLDALSPEQLVLTLLEAEPPHVL
hERb_v3      ERCGYRLVRRQRSADEQLHCAGKAKRSGGHAPRVRELLLDALSPEQLVLTLLEAEPPHVL
hERb_v2      ERCGYRLVRRQRSADEQLHCAGKAKRSGGHAPRVRELLLDALSPEQLVLTLLEAEPPHVL
hERb_5       ERCGYRLVRRQRSADEQLHCAGKAKRSGGHAPRVRELLLDALSPEQLVLTLLEAEPPHVL
             ******:*******.**:**  ****:.**:***:****::****************.**

mERb_v1      VSRPSMPFTEASMMMSLTKLADKELVHMIGWAKKIPGFVELSLLDQVRLLESCWMEVLMV
mERb_v2      VSRPSMPFTEASMMMSLTKLADKELVHMIGWAKKIPGFVELSLLDQVRLLESCWMEVLMV
hERb_v6      ISRPSAPFTEASMMMSLTKLADKELVHMISWAKKIPG-----------------------
hERb_v1      ISRPSAPFTEASMMMSLTKLADKELVHMISWAKKIPGFVELSLFDQVRLLESCWMEVLMM
hERb_v3      ISRPSAPFTEASMMMSLTKLADKELVHMISWAKKIPGFVELSLFDQVRLLESCWMEVLMM
hERb_v2      ISRPSAPFTEASMMMSLTKLADKELVHMISWAKKIPGFVELSLFDQVRLLESCWMEVLMM
hERb_5       ISRPSAPFTEASMMMSLTKLADKELVHMISWAKKIPGFVELSLFDQVRLLESCWMEVLMM
             :**** ***********************.*******

mERb_v1      GLMWRSIDHPGKLIFAPDLVLDRSSEDPHWHVAQTKSAVPRDEGKCVEGILEIFDMLLAT
mERb_v2      GLMWRSIDHPGKLIFAPDLVLD------------------RDEGKCVEGILEIFDMLLAT
hERb_v6      ------------------------------------------------------------
hERb_v1      GLMWRSIDHPGKLIFAPDLVLD------------------RDEGKCVEGILEIFDMLLAT
hERb_v3      GLMWRSIDHPGKLIFAPDLVLD------------------RDEGKCVEGILEIFDMLLAT
hERb_v2      GLMWRSIDHPGKLIFAPDLVLD------------------RDEGKCVEGILEIFDMLLAT
hERb_5       GLMWRSIDHPGKLIFAPDLVLD------------------RDEGKCVEGILEIFDMLLAT
```

```
mERb_v1      TARFRELKLQHKEYLCVKAMILLNSSMYPLATASQEAESSRKLTHLLNAVTDALVWVISK
mERb_v2      TARFRELKLQHKEYLCVKAMILLNSSMYPLATASQEAESSRKLTHLLNAVTDALVWVISK
hERb_v6      --------------------------MYPLVTATQDADSSRKLAHLLNAVTDALVWVIAK
hERb_v1      TSRFRELKLQHKEYLCVKAMILLNSSMYPLVTATQDADSSRKLAHLLNAVTDALVWVIAK
hERb_v3      TSRFRELKLQHKEYLCVKAMILLNSSMYPLVTATQDADSSRKLAHLLNAVTDALVWVIAK
hERb_v2      TSRFRELKLQHKEYLCVKAMILLNSSMYPLVTATQDADSSRKLAHLLNAVTDALVWVIAK
hERb_5       TSRFRELKLQHKEYLCVKAMILLNSSMYPLVTATQDADSSRKLAHLLNAVTDALVWVIAK
                                       ****.**:*:*:*****:**************:*

mERb_v1      SGISSQQQSVRLANLLMLLSHVRHISNKGMEHL---LSMKCKNVVPVYDLLLEMLNAHTL
mERb_v2      SGISSQQQSVRLANLLMLLSHVRHISNKGMEHL---LSMKCKNVVPVYDLLLEMLNAHTL
hERb_v6      SGISSQQQSMRLANLLMLLSHVRHASNKGMEHL---LNMKCKNVVPVYDLLLEMLNAHVL
hERb_v1      SGISSQQQSMRLANLLMLLSHVRHASNKGMEHL---LNMKCKNVVPVYDLLLEMLNAHVL
hERb_v3      SGISSQQQSMRLANLLMLLSHVRHARWGEKQFIHLKLS----------------------
hERb_v2      SGISSQQQSMRLANLLMLLSHVRHARAEKASQTLTSFGMKMETLLPEATMEQ--------
hERb_5       SGISSQQQSMRLANLLMLLSHVRHARSCVYK-----------------------------
             *********:**************      .

mERb_v1      RGYKSSISGSECCSTEDSKSKEGSQNLQSQ
mERb_v2      RGYKSSISGSECCSTEDSKSKEGSQNLQSQ
hERb_v6      RGCKSSITGSECSPAEDSKSKEGSQNPQSQ
hERb_v1      RGCKSSITGSECSPAEDSKSKEGSQNPQSQ
hERb_v3      ------------------------------
hERb_v2      ------------------------------
hERb_5       ------------------------------
```
