# Supplementary figures and images for: Environmental Xenoestrogens Super-Activate a Variant Murine ER Beta in Cholangiocytes
Source: Toxicol Sci. 2016 Dec 24;156(1):54–71. doi: 10.1093/toxsci/kfw234 (PMC5356623; doi:10.1093/toxsci/kfw234)

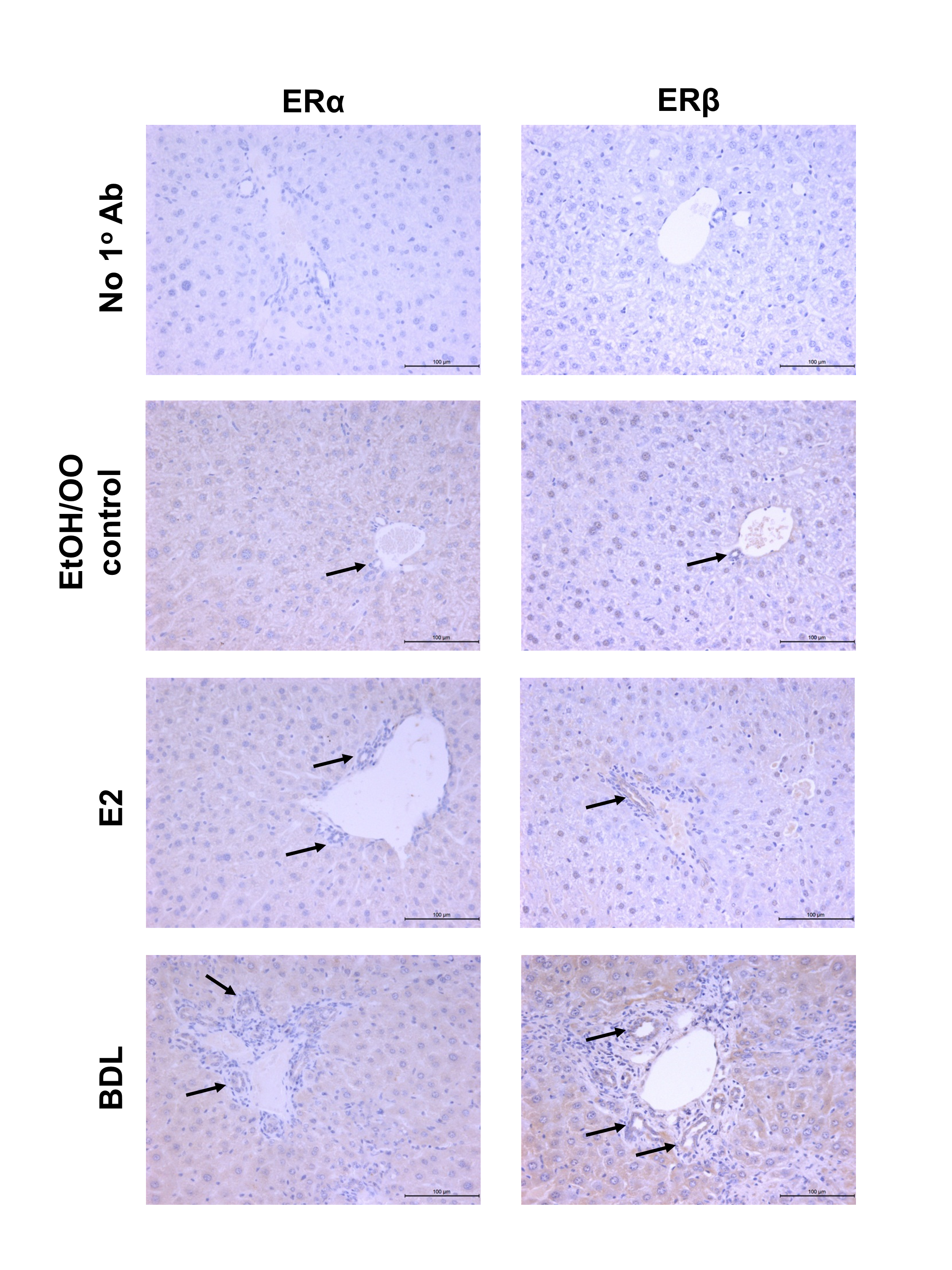

Supplement: Supplementary Data [file kfw234_Supp.zip › toxsci-16-0476-File014.TIF]

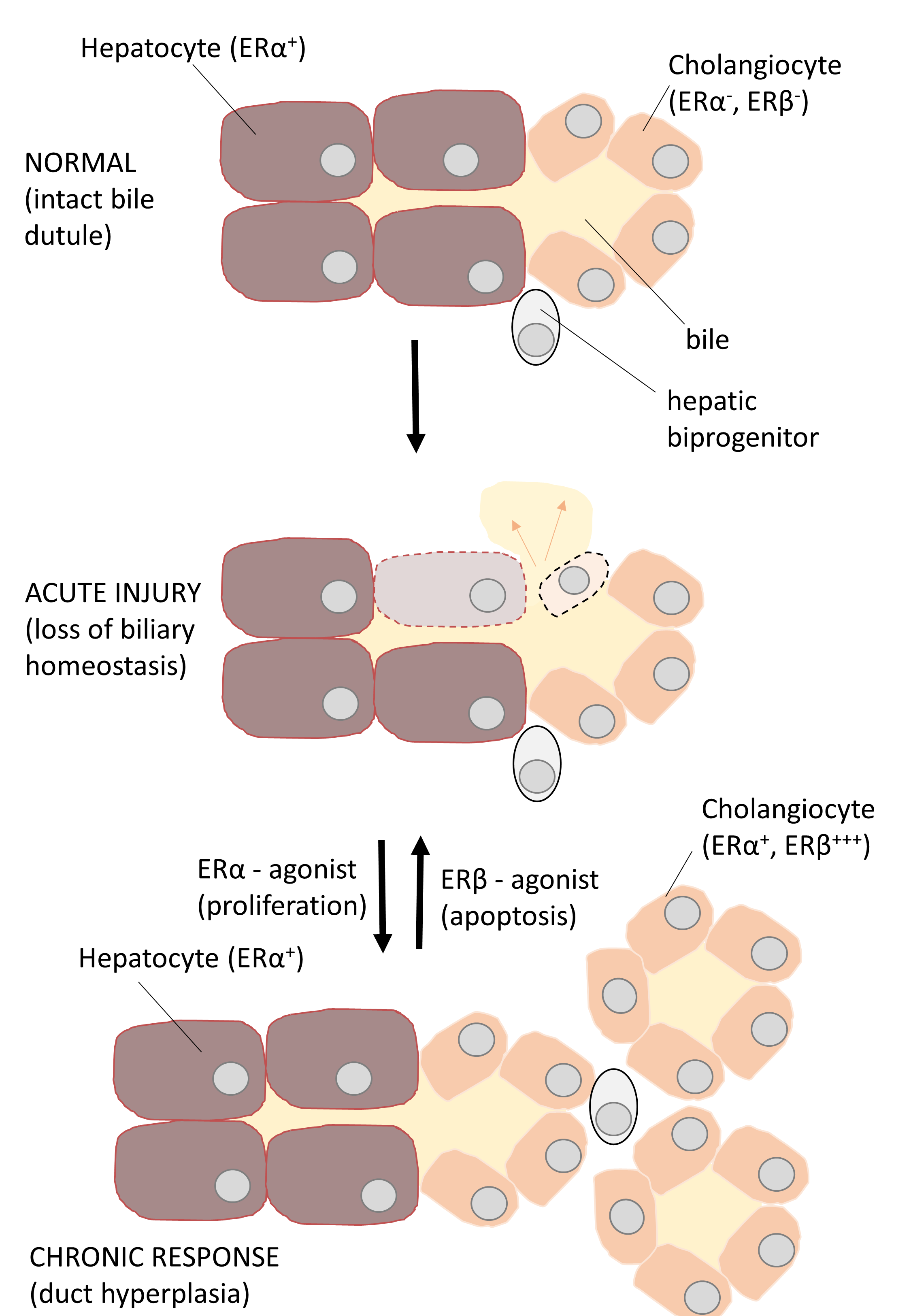

Supplement: Supplementary Data [file kfw234_Supp.zip › toxsci-16-0476-File015.TIF]
